# Probabilities of reaching required diffusion of granular energy technologies in European countries

**DOI:** 10.1016/j.isci.2025.111825

**Published:** 2025-01-16

**Authors:** Nik Zielonka, Evelina Trutnevyte

**Affiliations:** 1 Renewable Energy Systems, Institute for Environmental Sciences (ISE), Section of Earth and Environmental Sciences, University of Geneva, Geneva, Switzerland

**Keywords:** Engineering, Energy management, Energy Modelling, Energy Systems

## Abstract

To support discussions around the feasibility of the energy transition, we present probabilistic projections of the expected baseline diffusion of solar photovoltaics, wind power, biogases, heat pumps, and low-carbon passenger vehicles in 39 European countries for 2023–2050 under current contextual conditions. We then compare the projected capacities against those required for reaching net-zero greenhouse gas emissions by 2050. We create our projections using 72 different variants of diffusion models that we validate using hindcasting (2012–2022) and weight according to their performance. We find a notable expected growth in solar photovoltaics, heat pumps, and battery electric vehicles in the majority of European countries. However, most countries are unlikely to reach the required capacities for net-zero emissions of wind power and biogases with high confidence, and battery electric vehicles with lower confidence. Probabilities and confidence levels of reaching required capacities of solar photovoltaics and heat pumps vary the most between countries.

## Highlights

• Diffusion of granular energy technologies in Europe is analyzed probabilistically
• A notable growth in solar PV, heat pumps, and electric vehicles is expected
• Most countries will likely miss required capacities of biogases, wind power, and BEVs
• Probabilities of reaching required capacities of solar PV and heat pumps vary the most

## Graphical abstract

**Figure:**
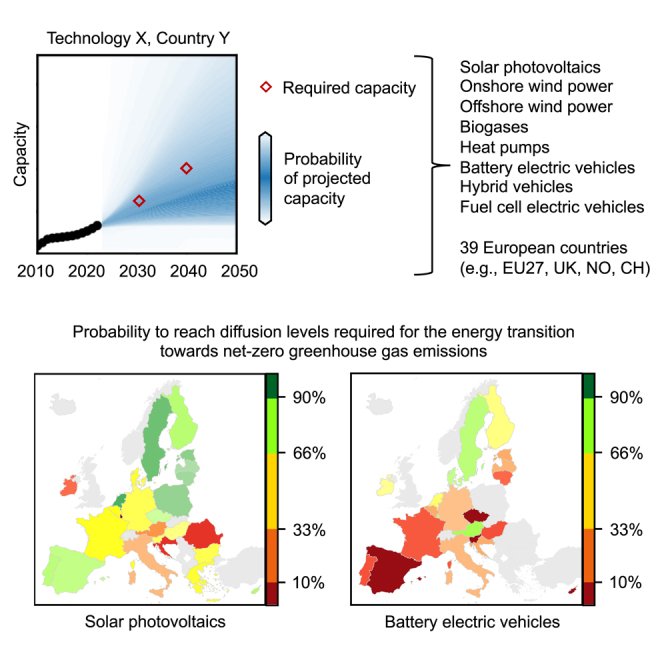

## Introduction

Granular energy technologies, which are at a more individual rather than institutional level,^1^ such as solar photovoltaics (PV), heat pumps or electric vehicles, are expected to play a key role for the energy transition in Europe and elsewhere.^2^^,^^3^^,^^4^ Due to comparatively small, modular capacities, lower risk and investment barriers, these technologies can diffuse faster than centralized technologies and can thereby accelerate the energy transition.^1^^,^^5^ Consequently, their diffusion is more dynamic, complex, and dependent on diverse non-technical factors that vary across countries,^3^^,^^4^ resulting in higher variations that make the growth of these technologies, particularly hard to project. Uncertainties increase even further if the technologies are at an early phase of diffusion and the availability of historical time series data are limited,^6^^,^^7^ which for now often applies to granular energy technologies.^8^^,^^9^^,^^10^

Projections of future diffusion of energy technologies are key for policymaking and infrastructure planning.^2^^,^^4^ Currently, projections oftentimes describe optimal pathways that energy system models determine for specific techno-economic, environmental, and policy conditions.^2^^,^^3^^,^^11^ Although some modelers aim to create not only conditional, exploratory scenarios, but also projections that are as realistic as possible from today’s point of view, they often fail to capture the actual future state of an energy system.^12^^,^^13^^,^^14^ Hence, recent research increasingly explores ways to conceptualize and quantify real-world feasibility of future energy projections.^5^^,^^15^^,^^16^ One way to model feasible projections is to adopt probabilistic methods by empirically learning from historical data.^6^^,^^17^^,^^18^ Such probabilistic projections do not describe discrete future scenarios anymore, but rather a range of possible future outcomes and thereby combine predictive thinking with in-depth uncertainty quantification.^18^^,^^19^^,^^20^ In addition to informing about probabilities and uncertainties that can help policy discussions and decision-making,^21^^,^^22^^,^^23^ probabilistic projections can also provide projections with higher real-world accuracy than deterministic methods.^6^ Earlier studies made first efforts in creating probabilistic projections for granular energy technologies in some European countries,^18^^,^^24^ but without comprehensively applying and testing a suite of relevant diffusion models and without comparing projected and required technology capacities to inform policymaking.

While there is a wide range of methods to create probabilistic projections, their use for modeling energy technology diffusion is still limited. For instance, Bayesian, regression, or system dynamics models rely on the availability of vast datasets that help to predict a quantity using conditional probabilities.^25^^,^^26^^,^^27^ Autoregressive models and neural networks rely on the availability of long time series data with repeating patterns to learn from.^28^^,^^29^ The use of empirical prediction intervals,^20^^,^^30^^,^^31^ where hindcasting errors between the projections and historically observed values are used to quantify the future uncertainty intervals, also requires sufficiently long time series data. Such error-based methods also mostly create symmetric intervals and only show uncertainty bounds without probabilities. Alternatively, many projections use random sampling, like Monte Carlo simulations, to generate a range of possible projections.^10^^,^^24^^,^^32^ Assigning ranges and probability distributions to parameters from random sampling is a challenging or even impossible task due to limited knowledge.^33^ Moreover, the generated forward-looking projections are rarely evaluated based on their accuracy and uncertainty performance. Validation is key to close the gap between the modeled projections and the feasible real-world transitions^13^^,^^14^ and it is particularly crucial when uncertainty intervals are narrow or the projection model is prone to overfitting, e.g., S-curve models.^6^^,^^34^ Thus, Zielonka et al.^6^ have recently combined sampling with hindcasting-based model validation to create probabilistic projections of granular energy technologies at a national and sub-national scale, but applied this approach only for three technologies in one country (Switzerland).

In this work, we investigate how granular energy technologies can be expected to grow in European countries until 2050 under current dynamics and contextual conditions, as well as the associated probabilities and uncertainties. Our methodology results in probabilistic projections that depict non-linear baseline trends that derive from past and current dynamics^35^ up to the last year of historical data used for model training. Historical time series of capacity diffusion encapsulate various factors like rates of sales and deployment, contextual conditions like policies and technological and socio-economic factors, as well as disruptive changes like economic crises, as commonly outlined in literature^34^^,^^36^ on diffusion curves. The probabilistic density intervals of the projections are hence derived from uncertainties in dynamics and diffusion curves (see STAR Methods) and cover the potential ranges of future growth that is expected under current dynamics and contextual conditions. We then compare this expected baseline growth against the levels that are required for the energy transition toward net-zero greenhouse gas emissions by 2050. Our probabilistic projections hence show if additional policy efforts are needed to increase the feasibility of net-zero emissions scenarios and are by design different from conditional scenarios and forecasts (see STAR Methods).

We investigate eight granular energy technologies at national level in 39 European countries (Table S1), including four electricity generation technologies (solar PV, onshore and offshore wind power, and biogases including biomass and organic waste), one heating technology (heat pumps), and three types of passenger vehicles (battery electric vehicles [BEVs], hybrid vehicles, and fuel cell electric vehicles [FCEVs]). We choose these technologies because consistent historical capacity data are available with non-static time series for the last five years in at least five countries to allow the creation and hindcasting-based validation of projections. All the eight technologies can be considered granular on the granular-lumpy continuum of Wilson et al.^1^ since all these technologies have modular units and unit sizes. As the degree of granularity differs across technologies, thereby influencing their diffusion, our projections and analyses are independent for each technology. The large scale of our study with eight technologies in 39 countries enables us to provide a comprehensive overview of the diffusion across Europe, while it consequently limits the extent by which we can provide case-specific interpretations and discussions that are beyond the scope of this work (see limitations of the study).

Provided that historical time series data are available, for each technology and country, we create and validate up to 72 different probabilistic projections (see STAR Methods) from a combination of six different S-curve models,^6^ three variants of near-optimal differential evolution (NODE) with different assumptions on growth rates, and four historical interval lengths, using a new approach called PROWIDE (see STAR Methods) that we extend from Zielonka et al.^6^ Using hindcasting-based validation of the projection performance between the years 2012 and 2022, we weight and combine the 72 projections for each technology and country to create probabilistic projections until 2050 (see STAR Methods). Through weighting, we only include the best performing models and filter out projections that overfitted historical data and projected inaccurate future quantities in out-of-sample testing. We then also validate our final weighted projections and compare our probabilistic projections against the installed capacities of these technologies required for the European energy transition at national level according to the scenarios of the European Green Deal policy package “Fit for 55”^37^^,^^38^ and the Ten Year Network Development Plan (TYNDP)^39^ in 2030 and 2040 (see STAR Methods). We focus on informing national decision-makers and therefore present our results primarily in absolute numbers for each country.

We show that the numbers of electric vehicles will most likely increase notably under the current trends in most European countries, although uncertainties are high. While solar PV and heat pumps show a notable growth in most countries, the probability of reaching the respective capacities required for the transition toward net-zero varies between countries. Future capacities of BEVs, biogas, and wind power will likely remain lower than the estimated transition requires with high confidence.

## Results

### There is no single best model variant for all technologies and countries

From the methodological point of view, we find that the 72 model variants that we use for our projections exhibit hindcasting performance that varies notably across technologies and countries (Figures S1–S6). While there is no single best model variant that scores consistently better than others, the variants using the logistic S-curve model consistently perform lowest on average. Importantly, an out-of-sample validation (2017–2022) of the final weighted probabilistic projections shows consistently higher accuracy and more reliable and accurate ranges of probabilistic density intervals than any of the single model variants (Figures S7–S12), pointing to the advantage of scoring and weighting models in PROWIDE. The out-of-sample capacities of projected technologies lie statistically significantly with high probability within the probabilistic density intervals (Table S2) and most often even within the interquartile range (Figure S13). While the out-of-sample capacities of wind power are comparatively close to the medians of our projections, the capacities of biogases tend to be lower and the capacities of solar PV, heat pumps, BEVs, and hybrid vehicles tend to be higher than the median. Nonetheless, the absolute percentage deviation between the medians and out-of-sample capacities are comparatively low for onshore wind power, biogases, and heat pumps (Figure S14). Furthermore, the deviation of 1-year-ahead capacities from the historical values decreases over time for electric vehicles, indicating that the projections adapt to changes in the diffusion dynamics over time.

### Notable expected growth in solar PV, heat pumps, and electric vehicles

**Figure 1:**
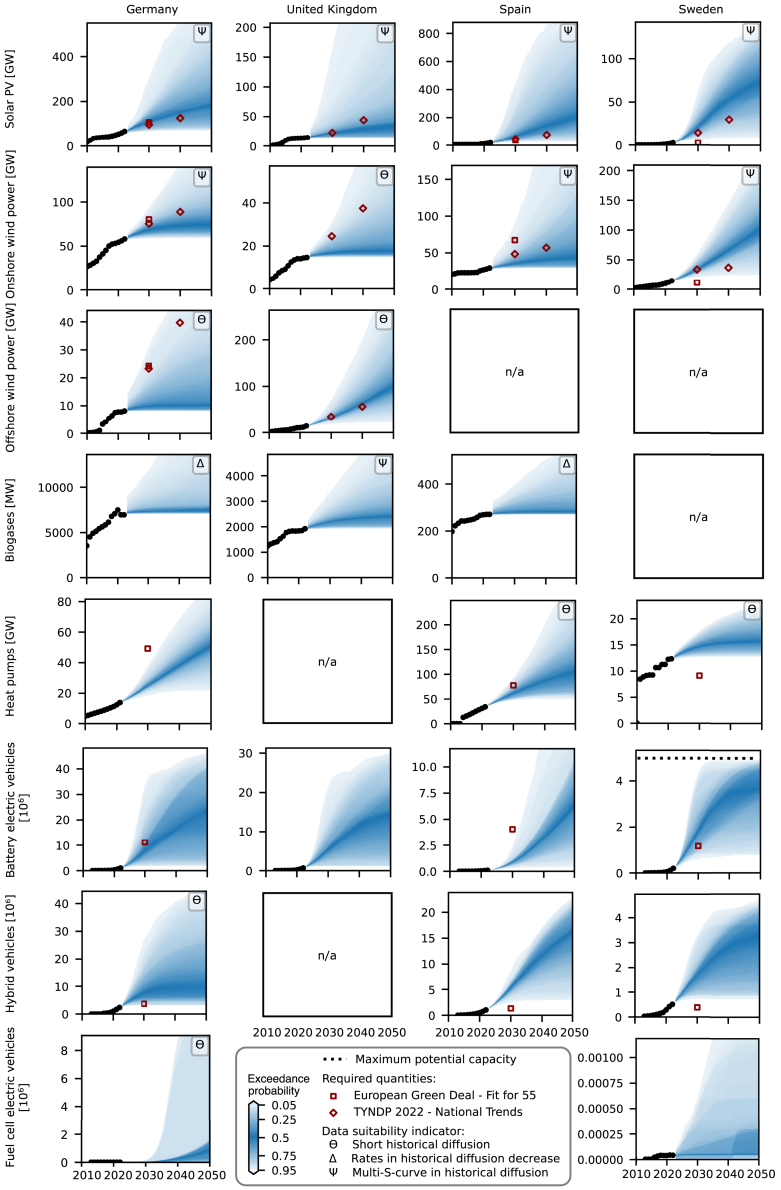
Probabilistic projections under current dynamics and contextual conditions for all eight investigated technologies in Germany, the United Kingdom, Spain, and Sweden, including training data until 2022, respectively, 2021 for heat pumps Training data starts in 1990, respectively, 2013 for electric vehicles. The probabilistic density intervals show the probability that a quantity will be reached or exceeded if the diffusion follows current trends. The color gradient describes the quantiles of the projected capacities: the darker the color, the closer the capacity is to the median. The black dots show historical capacities and the black dotted lines, if visible, the maximum potential capacity that a country can install (see STAR Methods). The red squares and diamonds show required quantities for the energy transition, estimated in scenarios of European Commission^37^^,^^38^ and Ten Year Network Development Plan^39^ that are consistent with the European Green Deal policy package “Fit for 55”, and national energy and climate policies (see STAR Methods). Required quantities may be invisible if they are larger than the upper limit of the vertical axes of the projections. The Greek letters indicate a qualitative assessment on the suitability of the historical time series data for projecting probabilistic growth (see STAR Methods), where a short historical diffusion (Θ) results in less reliable projections as empirical testing is comparatively short, decreasing historical diffusion rates (Δ) may result in early saturation, and a multi-S-curve pattern (Ψ) may lead to an underestimation of future growth. Figures S15–S24 provide projections for the remaining countries.

Our baseline probabilistic projections reveal a notable variation among the European countries in terms of which granular energy technologies are more likely to grow and at what rate under current dynamics and contextual conditions (Figure 1). For example, in Germany, the only country with projections for all eight investigated technologies, solar PV will most likely double in capacity by 2050, heat pumps will triple (see medians in Figure 1) if the diffusion follows current trends. In contrast, the capacities of biogases and offshore wind power will remain relatively low. Unless further promoted or restricted, the numbers of BEVs can be expected to grow similarly as the numbers of hybrid vehicles, whereas the numbers of FCEVs will remain comparatively low over the next decades. This relation between electric vehicles can be observed for almost all European countries (Figure S15–S24). The trend in solar PV capacities is also representative for most European countries, except for the United Kingdom (UK), for instance. Here, however, offshore wind power will likely grow faster than in other investigated countries under the current dynamics. The probabilistic projections for biogases and onshore wind power show a diffuse picture where, for example, biogases increase in Cyprus, Greece, and Turkey, and onshore wind power in Belgium, Finland, Ireland, and Netherlands, while both capacities remain close to current levels in Luxembourg, Portugal, and Spain. For heat pumps, the projections estimate a notable baseline increase in capacity for most countries until 2050, except for Norway and Sweden, where the use of heat pumps is already high^40^^,^^41^ and their diffusion slowed down in recent years.

**Figure 2:**
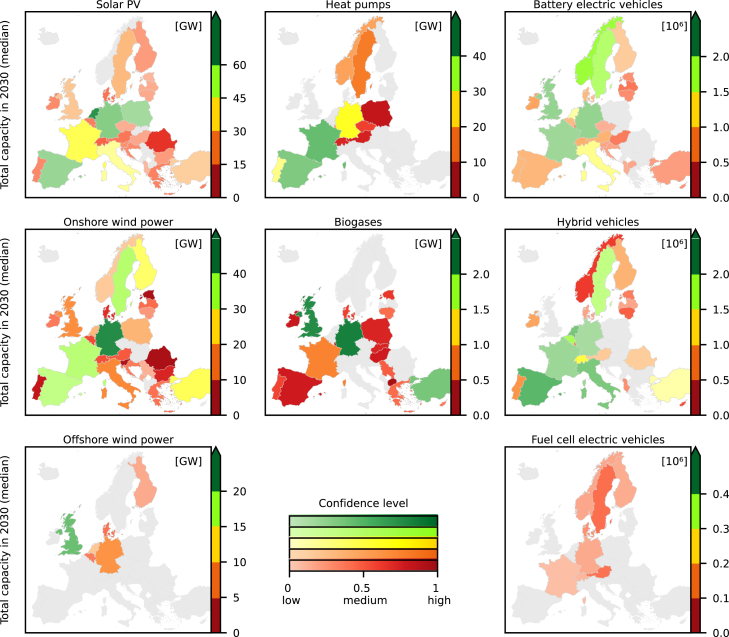
Distribution of median capacities of all eight investigated technologies across Europe in 2030 The colors group countries according to the total capacity estimated by the median in the probabilistic projections. The color gradient indicates the confidence level: the darker the color, the higher the confidence. The confidence level describes the width of a probabilistic projection and is defined as the share of quantiles that covers the range of ±25% from the projected median quantity (see STAR Methods). The lower the share, the broader is the probabilistic projection. Countries in gray have no projection. See Figures S25 and S26 for the years 2040 and 2050. See Figures S27–S29 for capacities per capita.

The projected baseline diffusion of granular energy technologies highlights how European countries favor specific technologies and how the capacities will most likely spread across Europe under current dynamics and contextual conditions (Figures 2 and S25 and S26). Based on these conditions, Germany, the country with the largest number of inhabitants,^42^ will most likely be among the countries with the largest installed capacities of solar PV, wind power, and biogases and the largest number of electric vehicles in the next decades. In the UK, the comparatively small projected growth in solar PV but high growth in offshore wind power reflects in absolute values of installed capacities. This relation is compatible with the capacity factors in the UK which are comparatively low for solar PV and high for wind power.^43^ The offshore wind power capacities also remain one of the highest in the UK when scaled by capita, while the distribution of onshore wind power and solar PV across Europe becomes evener (Figures S27–S29). In absolute capacities, the distribution of onshore wind power and solar PV is diffuse across Europe. Besides Germany, highest capacities are projected for solar PV in France, Netherlands, Poland, and Spain, and for onshore wind power in the Nordic countries, France, Spain, and Turkey. Sweden stands out with high shares per capita for solar PV, onshore wind power, BEVs and hybrid vehicles. Both in relative and absolute terms, the highest capacities of heat pumps are projected in France, followed by Portugal and Spain, and of biogases in Germany, Turkey, and the UK. All other countries will likely have low capacities of both technologies if current trends continue. Like with Germany, the highest projected numbers of electric vehicles are where most people live, e.g., in the UK (BEVs), Spain, France, and Italy (hybrid). Besides Sweden, the numbers per capita are highest for Norway and Switzerland (BEVs), and Belgium, Luxembourg, Italy, Spain, and Switzerland (hybrid).

While the magnitude by which a technology is projected to grow varies notably between technologies and countries, our probabilistic projections most often show similar levels of confidence on the projected capacities for the same technology (Figure 2). High confidence means that the inner probabilistic density intervals of a projection are sharp, whereas the overall range of uncertainty can remain high, e.g., offshore wind power in the UK (Figures 1 and 2). For most countries, the confidence is comparatively high in the projections of biogases and onshore wind power and medium in the projections of solar PV, offshore wind power, and heat pumps (Figure 2). Compared to the other technologies, the historical time series of electric vehicles are short and hence lead to higher uncertainties and consistently lower confidence. The historical time series also reflect the way that the confidence reduces the further away the projected year is from today. For instance, the historical diffusion of heat pumps is relatively steady in most countries and the probabilistic projections hence form a relatively regular cone (Figures 1 and S15–S24). In contrast, if the historical diffusion is unsteady, the width of the probabilistic density intervals evolves rapidly and into irregular shapes, e.g., biogases and wind power.

### Most countries will unlikely reach required quantities of wind power, biogases, and battery electric vehicles under current trends

**Figure 3:**
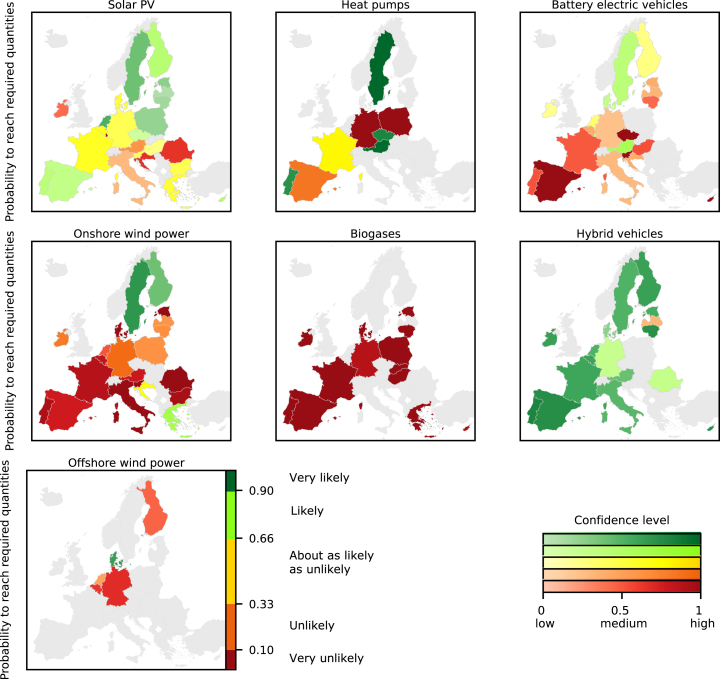
Map showing the exceedance probability of countries to reach required quantities in 2030 The required quantities are published in or derived from scenarios that are consistent with the European Green Deal policy package “Fit for 55” by the European Commission^37^^,^^38^ (see STAR Methods). The colors group all countries according to the level of probability and translate it into qualitative statements on likelihoods in line with earlier IPCC guidelines.^44^ The likelihoods are conditional to the current dynamics and contextual conditions until the last training year. The color gradient indicates the confidence level: the darker the color, the higher the confidence. The confidence level describes the width of a probabilistic projection and is defined as the share of quantiles that covers the range of ±25% than the projected median quantity (see STAR Methods). The lower the share, the broader is the probabilistic projection. Countries in gray have either no projection or no values of required quantities. See Figure S30 for the probabilities to reach required quantities from the Ten Year Network Development Plan in 2030 and 2040.

When comparing the probabilistic projections against capacities that European countries need in 2030 and 2040 for the transition toward net-zero greenhouse gas emissions according to “Fit for 55”^37^^,^^38^ and TYNDP^39^ scenarios (see STAR Methods), we find that most countries are unlikely to reach the required capacities of BEVs, biogases, and wind power, while most will likely reach the numbers for hybrid vehicles under current dynamics and contextual conditions (Figures 3 and S30). For solar PV and heat pumps, the probabilities vary the most among countries. For solar PV, countries in north-eastern Europe, Netherlands, Portugal, and Spain will likely reach the required capacities, while countries from southern Europe and Ireland will likely miss them. For heat pumps, Austria, Czech Republic, Portugal, and Sweden will likely reach the required capacities, while Germany, Poland, and Spain will likely miss them. In most cases, our findings are consistent with the trends from both considered transition scenarios of “Fit for 55” and TYNDP. However, for some countries where the estimated capacities required for the transition diverge notably between “Fit for 55” and TYNDP, the probabilities of reaching these required capacities can diverge too. For example, “Fit for 55” provides a comparatively low value for offshore wind power in Denmark that our projected baseline diffusion will very likely reach by 2030, whereas Denmark is unlikely to reach the high value from TYNDP (Figure S17). Consequently, drawing universal conclusions on the feasibility of the energy transition is limited in such cases. At the same time, the required quantities for some countries are comparatively low and thus require only small growth in capacities. For heat pumps and hybrid vehicles in Sweden, for example, the capacities required for the transition according to “Fit for 55” are even lower than the current capacities (Figure 1), which challenges assumptions in our estimation of required capacities for Sweden (see Data).

The confidence for reaching or missing the required quantities for the energy transition is transferable from the confidence on the projected quantities for each technology and country (Figures 2 and 3). Consistently, confidence is generally high for biogases and wind power, and comparatively low for BEVs. Also, the confidence levels related to reaching the required capacities of heat pumps are rather high, while confidence levels related to solar PV vary the most between countries. Overall, the comparison against required quantities shows that if the probability of reaching a required quantity is either very high or very low, confidence of reaching or missing a required quantity is high. With medium levels of probability, confidence is more diffuse.

## Discussion

Our baseline probabilistic projections of granular energy technologies under current dynamics and contextual conditions in Europe reveal different types of diffusion that can roughly be divided into three groups: (1) high growth with low levels of confidence, (2) low growth with medium to high confidence, and (3) medium to high growth with medium confidence. Although it varies country by country in which group the projections of each technology fall, there is a tendency for BEVs and hybrid vehicles to fall into the first group, biogases and wind power into the second, and solar PV and heat pumps into the last. Reasons for this tendency are manifold. For example, diffusion of biogases and wind power has recently slowed down in many countries while the diffusion of electric vehicles gathered speed. Nevertheless, the tendency from our findings still indicates that even more granular technologies like electric vehicles and solar PV tend to diffuse faster with lower confidence than less granular technologies, as presented in literature.^1^^,^^5^

The projected growth in granular energy technologies will not only lead to an uneven spatial distribution of capacities across Europe but also to uneven extent and confidence with which countries will likely reach or miss the levels required for the transition toward net-zero emissions under current dynamics and contextual conditions. Only the diffusion of hybrid vehicles is an exception as all countries will likely reach the required numbers, except for Latvia, but required numbers for hybrid vehicles are also close to current stocks. While this finding shows that European countries increasingly switch toward low-emission vehicles, BEVs must in the future take an even higher share and replace hybrid, petrol, and diesel vehicles to reach the required numbers for the net-zero emissions system. The new regulation^45^ of the European Union that only allows new registrations of emission-free passenger vehicles from 2035 on would further speed up the uptake of BEVs above our baseline projections. However, European countries should carefully monitor the future diffusion as confidence on the projected numbers is comparatively low and political targets^46^ can differ from the required quantities for “Fit for 55” scenarios, e.g., Figure S31. Almost all countries that need higher capacities of biogases and wind power for the transition must intensify their efforts toward higher diffusion. For solar PV, southern European countries, except for Portugal, Spain, and Turkey, are particularly the ones that should intensify their efforts not only since they will likely miss the required capacities for the net-zero targets but also as they have higher capacity factors^43^ and can therefore profit from higher PV output and thereby cheaper PV electricity than northern European countries. The conclusions for heat pumps are less evident since the required capacities for the transition are less clearly defined, if at all, and can lay close or even below current values.

Probabilistic projections should always use and test multiple S-curve models as the hindcasting performance in terms of accuracy and uncertainty varies highly from case to case, as also shown in previous work.^6^ When computational or other constraints limit the number of S-curve models that can be used, modelers should provide compelling reasons for their model selection. In contrast to common practice,^34^^,^^47^^,^^48^ the logistic model should be first model to be removed. Then, modelers can limit computational costs in many cases by selecting one NODE variant or by reducing the historical interval length they use for curve fitting. Reducing the historical interval length in training has the additional advantage of allowing diffusion models more flexibility in accounting for recent contextual changes and projecting more diverse levels of diffusion while keeping the same level of performance of the projections. Instead of comparing many model variants, modelers could then focus more on comparing diffusion models that are substantially different from each other and thereby create performance gain and robustness of the projections. Likewise, modelers could learn from applying our approach to alternative technologies in later diffusion phases to further improve the projections. Our comparison of 72 model variants using hindcasting can only show which variants perform comparatively better for the investigated technologies and countries and assign high weights to these variants accordingly, which could create a risk of overconfidence in these models. Therefore, to assess the performance gain of models, modelers should always compare all models using out-of-sample performance indicators as in Figures S1–S6, and not using weights only.

In terms of policy implications, our probabilistic baseline projections emphasize that decision-makers in European countries must consider additional policies to speed up the diffusion of the investigated granular energy technologies to reach the levels required for energy systems with net-zero emissions, especially for wind power, biogases, BEVs, and in some countries also solar PV and heat pumps. Our projections provide country-specific information on probabilities and confidence of future technology growth and can thereby direct attention to the most problematic issues and support policy debates and decision-making. Our findings are generally in line with some earlier projections and examinations in literature,^49^^,^^50^ although we provide more detailed, quantitative evidence along with probabilities. As the next step, decision-makers could investigate alternative technology mixes that advance the energy transition if supported by studies that combine our probabilistic technology diffusion projections with different scenarios of the whole energy system. Doing so would help to explore different energy futures while having information on which futures are more feasible than others and what the associated uncertainties are.

### Limitations of the study

From the methodological point of view, our approach is transferable to alternative technologies and regions, while there are two main avenues for future work. First, our approach for now does not analyze how contextual factors like policy decisions, technological developments or societal complexities drive the diffusion. Having said that, recent changes in contextual factors may show no direct effect on the projected diffusion due to lag^51^ and if there are no comparable changes in the historical time series data. We, therefore, recommend updating projections annually using the updated time series that include the most recent changes in most recent diffusion dynamics and contexts. Our projections including annual updates are available on Zenodo: https://doi.org/10.5281/zenodo.10532786.^52^ Accordingly, reasons remain unclear why specific countries will likely reach the technology levels required for the transition while others will not. Our projections disallow to judge whether reaching or missing required levels is due to, for instance, the (in-)existence of past and current policies, efforts of decision-makers, or other contextual conditions. Mapping our results to a set of contexts could help explaining differences between countries and technologies, and further increase the case-specific accountability of our projections.

It also remains unclear how changes in contextual conditions and diffusion dynamics may disrupt the diffusion and eventually lead to other capacities that our projections estimate (Figures S13 and S14). While the possibility of testing such changes in hindcasting is inherently limited^35^ as we investigate a non-isolatable, non-repeatable, and permanently changing system in which the diffusion takes place, we can only show that our projections cover such changes more reliably and accurately than the 72 alternative model variants (Figures S7–S12). Alternative methods that explicitly model decisions in the diffusion of technologies, such as system dynamics, agent-based or optimization models,^27^^,^^28^^,^^53^ could add complementary insights to the understanding of diffusion processes and their alignment or deviation from our projections. The investigation of policies and contextual factors in hindsight and in forward-looking sense^51^^,^^54^^,^^55^ should hence ideally be coupled with probabilistic projections to provide a more detailed understanding of the feasibility of reaching pre-defined levels of technology diffusion in the future and the relevance of contextual factors, policies or disruptive events in history and future. Such investigation could include real data on decisions and factors, or scenarios on a wide range of future developments.^56^

Second, our projections neglect any system effects and correlations in which the diffusion of one technology accelerates or inhibits the diffusion of another one, for instance, due to co-adoption, technology competition or other correlations across technologies or countries. Investigations of such effects on future energy systems are evolving^57^^,^^58^^,^^59^ and remain an important subject of future work in the field of probabilistic projections.^17^^,^^60^

## Resource availability

### Lead contact

Further information and requests for resources and materials should be addressed to Nik Zielonka (Nik.Zielonka@unige.ch).

### Materials availability

Materials and methods are described in the STAR Methods.

### Data and code availability

• Data: all probabilistic projections created and presented in this study are freely available on Zenodo: https://doi.org/10.5281/zenodo.10532786,^52^ including annual updates using the latest statistics on capacity diffusion. The data section in the STAR Methods references all data we use to create our projections.
• Code: we describe all steps to replicate our codes in the STAR Methods.
• Additional information: any additional information required to reanalyze the data reported in this paper is available from the lead contact upon request.

## Acknowledgments

This work received funding from (i) the partnership between University of Geneva and Services Industriels de Genève, (ii) the Swiss State Secretariat for Education, Research, and Innovation SEFRI for the project IAM COMPACT “Expanding Integrated Assessment Modelling: Comprehensive and Comprehensible Science for Sustainable, Co-Created Climate Action” (project no. 101056306), (iii) the Swiss Federal Office of Energy as part of the SWEET consortium SURE, (iv) and the Swiss National Science Foundation Eccellenza grant as part of the project “Accuracy of long-range national energy projections” (grant no. 186834). The computations were performed at the University of Geneva using Baobab High Performance Computing service. The authors bear sole responsibility for the conclusions and the results.

## Author contributions

Conceptualization, methodology, investigation, formal analysis, data curation, software, visualization, writing (original draft), N.Z.; conceptualization, supervision, funding acquisition, writing (review and editing), E.T.

## Declaration of interests

The authors declare no competing interests.

## STAR★Methods

### Key resources table

**Table:** 

REAGENT or RESOURCE	SOURCE	IDENTIFIER
**Deposited data**		

Original dataset	This paper / Zenodo	https://doi.org/10.5281/zenodo.105327876

**Software and algorithms**		

Excel for Mac 16.90.2	Microsoft	https://learn.microsoft.com/en-us/officeupdates/release-notes-office-for-mac
Python 3.9.12	Python Software Foundation	https://www.python.org/downloads/release/python-3912/

### Experimental model and study participant details

This study did not use any experimental model with study participants.

### Method details

#### Creation and validation of probabilistic projections – PROWIDE approach

We create our probabilistic projections for each technology and country by applying an extended version of the four-step approach of Zielonka et al.^6^ that we call PROWIDE (PRObabilistic projections using Weighted models and Iterative hindcasting for empirically based DEnsity intervals). The original approach^6^ has been developed to model probabilistic projections at a subnational level and hence requires data from many regions to create meaningful projections. Tests of the previous approach have shown that it can project national-level diffusion too with comparable levels of accuracy, but it needs alternative ways to provide probabilities.^6^ Consequently, we have extended the approach and developed PROWIDE to create probabilistic projections based solely on historical time series data of the considered country.

The probabilistic projections that we develop are different from conditional, exploratory scenarios that depict what-if outcomes in the future and do not provide any information about the likelihood of these outcomes. Our projections are also different from forecasts because we do not follow predictive rationale and acknowledge substantial uncertainties. These uncertainties naturally increase in time, and at the same time, they are still narrower than the uncertainties from meta-reviews of existing what-if scenarios^56^^,^^61^ that go beyond trends of projections.

**Figure 4:**
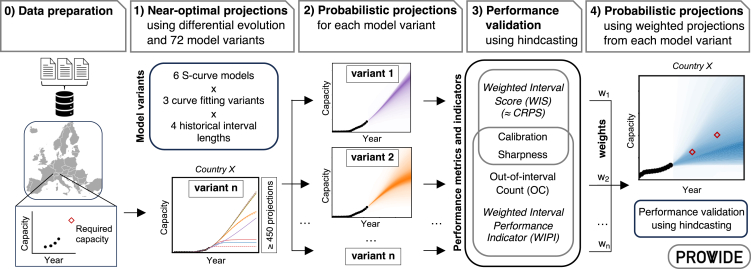
Flow chart of the PROWIDE (PRObabilistic projections using Weighted models and Iterative hindcasting for empirically based DEnsity intervals) approach, extended from Zielonka et al.,^6^ to create probabilistic projections of granular energy technology diffusion at national level Each hindcasting iteration repeats steps 1 and 2 using one share of the historical time series data for training and the remaining share of up to 10 years for validation. Calibration and sharpness are part of the weighted interval score that approximates the Continuous Ranked Probability Score (CRPS).^62^ The weighted interval performance indicator sums calibration, sharpness, and out-of-interval count. Each of the *n* = 72 model variants is a combination of one out of six S-curves, one out of three curve fitting variants, and one out of four historical interval lengths (see Supplemental Methods S1 and S2) and creates 450 or more deterministic projections that make up one probabilistic projection.

After data preparation (step 0), PROWIDE consists of four major steps (Figure 4) that we repeat separately for each technology and country pair. For the first steps, we exclude quasi-static and highly fluctuating historical time series as they do not record meaningful diffusions that we can project and test empirically. We include time series if at least three most recent capacities are non-zero, not more than five consecutive capacities are the same (three for electric vehicles), and there is no drop in the time series from one year to another by more than 30%. Consequently, the inclusion criteria reduce the number of technologies and countries that we analyze in our case study, but they have no effect on the projections for the remaining technologies and countries. More relaxed criteria would increase the number of technologies and countries but risk the creation of less reliable projections.

In step 1, we create deterministic projections for each technology and country using up to 72 model variants (see Supplemental Methods S1). Each variant is a combination of one S-curve model (Bass, Bertalanffy, Gompertz, logistic, Richards-4p, Richards-5p; see Supplemental Methods S2), one curve fitting variant that uses Near-Optimal Differential Evolution (NODE) to create projections with and without the consideration of historical diffusion rates (NODE, NODE-RR, NODE-DR), and a historical interval length (5 years, 10 years, 15 years, or full history). NODE is a method we developed for this study (see Supplemental Methods S1) that uses the metaheuristic approach of differential evolution^63^^,^^64^ to explore possible fits *f(a)* of an S-curve^6^ with parameters *a* to the historical capacities *y* of years *t* until it finds the optimal fit where the residual sum of squares between the fit and the historical capacities is minimized (Equation 1). Parameters *a* must lay within boundaries *c*_low_ and *c*_up_ (Supplemental Methods S2).

(Equation 1) $$\min\left(\sum_{i=1}^{t}{\left(y_{i}-f_{i}\left(\underline{a}\right)\right)}^{2}\right)subject\;to:{\underline{c}}_{low}\leq \underline{a}\leq {\underline{c}}_{up}$$

NODE iteratively repeats the differential evolution and randomly picks 450 or more fits near the optimum, resulting in 450 or more deterministic projections (see Supplemental Methods S1). By design, neither differential evolution nor the random picking in NODE require probability distributions of model input parameters, which contrasts common sampling methods like Monte Carlo.^32^ Step 1 is repeated for each model variant. We limit the number of deterministic projections to at the minimum 450 to save computational costs and since we find that the resulting projections are comparable to the ones with 200 projections that we create in prior tests of our approach.

In step 2, we calculate for each variant separately the quantiles *q* of all its 450 or more deterministic projections to describe probabilistic projections of each model variant. The quantiles translate into probabilities and confidence of a probabilistic projection. The exceedance probability *p*_*ex*_ describes the probability that a future quantity will be reached or exceeded and is the opposite of a quantile *q*:

(Equation 2) $$p_{ex}=1-q$$

The median (50% quantile) shows the center of a probabilistic projection at which it is equally likely that the observed value of the quantity will lay above or below. We define the level of confidence on a projected quantity as the width of the probabilistic density intervals that covers a ±25% deviation from the median of a projection. Thereby, the confidence reflects the sharpness of the probabilistic projection. The confidence can vary between 0, where both the upper and the lower bounds of the ±25% deviation lay between the same quantiles, and 1, where the width of the entire probabilistic projection is equal or smaller than the difference between the upper and the lower bound of the deviation. In line with earlier IPCC guidelines,^44^ we show increasing confidence with increasing strength of shading when comparing probabilistic projections between countries, e.g., Figures 2 and 3.

In step 3, we evaluate the probabilistic projections of all model variants separately for all technologies and countries using hindcasting where we compare our probabilistic projections against historical observations from 2012 to 2022. In each hindcasting iteration, we split the historical time series data into a training set with which we repeat steps 1 and 2, and an out-of-sample test set which we use to validate the created probabilistic projections using performance metrics. After each iteration, we remove one more year from the validation set and add it to the training set. As we do not pass any information on model performance back to the creation of projections in steps 1–2, each iteration is independent and clear from learning effects. We do hindcasting for the last ten years for the power generation technologies and heat pumps, and for the last five years for the electric vehicles. In hindcasting, we evaluate the probabilistic projections based on the unitless Weighted Interval Performance Indicator (WIPI).

The WIPI sums three performance metrics equally, that we normalize from 0 to 1. The first two metrics are calibration^62^^,^^65^ and sharpness^62^^,^^65^ that sum into the weighted interval score that approximates the continuous ranked probability score (CRPS)^62^ and are common metrics to score the performance of probabilistic projections and forecasts.^20^^,^^62^^,^^65^ Calibration evaluates the accuracy of a probabilistic projection to historically observed data, sharpness reflects the width of probabilistic density intervals. The third metric we add in the WIPI is out-of-interval count (OC)^66^ that counts historically observed values that lay outside the quantile range of 0.01 and 0.99 and thereby, adding to CRPS, penalizes projections in our performance validation that fail to cover the future diffusion of technologies in their probabilistic density intervals. Consequently, OC acts as a measure of reliability on the range of the probabilistic density intervals. The lower the penalties of sharpness, calibration, and OC are, the better the performance of the projections.

In step 4, we use the WIPI to weigh the probabilistic projections of all model variants separately for all technologies and countries and eventually create our final probabilistic projections through quantile averaging.^65^ Each model variant receives weights that are independent from all other weights it receives for other technologies and countries. We define the unitless weights *w* for each model variant using an equation adapted from Sun et al.^67^ and Zielonka et al.^6^:

(Equation 3) $$w=1/mean\left(WIPI^{2}\right)$$

Like for the 72 model variants, we also perform an out-of-sample validation of our final probabilistic projections using hindcasting (Figures S7–S12).

#### Data

##### Historical time series and potentials

To create our probabilistic projections we take historical time series of cumulative installed capacities for each technology and country (Table S1) from Eurostat.^8^^,^^9^^,^^68^ We fill data gaps for power generation technologies with values from IRENA,^69^ for battery electric vehicles in United Kingdom from SMMT,^70^ and for heat pumps in Switzerland from SFOE.^71^ Heat pumps include aerothermal, geothermal, and hydrothermal technologies. Hybrid vehicles include plug-in and full hybrid vehicles with petrol or diesel. Altogether, the historical time series range from 1990 to 2022 for power generation technologies, 1990–2021 for heat pumps, and 2013–2022 for electric vehicles. For each technology and country, we limit the potential of maximum installable capacities not to be exceeded in S-curve fitting using data from literature and Eurostat (see Supplemental Methods S3). We choose relatively high numbers from literature to reduce the influence of our choice on the fitting. The limits do not influence the projections unless the limits are reached by the S-curve fits. In contrast, if future shows that the limits reduce below the maximum of our probabilistic projections, users can cut the projections above the new limit.

To indicate the suitability of the historical time series data and thereby provide a qualitative assessment on the reliability of the probabilistic projections, we add one of the following labels to the projections. These data suitability label contrasts the confidence level of projections as it presents a quantitative indication of the width of a probabilistic projection, provided the data are considered suitable.

Θ “Short historical diffusion” if hindcasting is not possible for all years due to the exclusion criteria in step 1. Consequently, projections may be considered less reliable as empirical testing is comparatively short.

Δ “Rates in historical diffusion decrease” if the historical diffusion rates do not monotonically increase. Consequently, the diffusion models may interpret saturation and project no future growth.

Ψ “Multi-S-curve in historical diffusion” if the historical diffusion rates increase after a previous decrease. Consequently, the diffusion models may capture only the first growth phase and underestimate possible future growth unless the historical interval length is reduced enough to skip the first growth phase.

##### Technology quantities required for the energy transition

To assess whether the current diffusion of the investigated technologies is on track to reach future capacities required for the European transition to net-zero greenhouse gas emissions, we compare our projections against three values. The first value is the mean of three scenarios of the European Commission that are consistent with the European Green Deal policy package of reaching 55% reductions in greenhouse gas emissions in the European Union by 2030 (“Fit for 55”).^37^^,^^38^ Hence, these required capacities from scenarios are internally consistent across Europe, but do not represent individual political targets set by national governments. While we directly use the estimated net installed capacities^37^ for solar PV, wind power, and biogases, we derive the required national capacities of heat pumps and the numbers of BEVs and hybrid vehicles using a set of assumptions. We assume that the change in the final energy consumption of ambient heat^37^ from 2020 to 2030 approximates the factor by which the heat pump capacity needs to change. Multiplying this factor with the heat pump capacities^68^ of 2020, we get the required capacities for 2030. For BEVs and hybrids, we use the share of electricity in the national road transport^37^ to approximate the share of electric vehicles over all road vehicles^72^ as the share in non-passenger vehicles will remain comparatively negligible.^73^ We then scale the corresponding shares of national electric vehicles so that total required shares of BEVs and hybrids in all passenger vehicles of the European Union in 2030 are 15.7% and 5.2%,^38^ respectively. The second and third values of required quantities are estimates for 2030 and 2040 from the TYNDP scenario “National Trends”^39^ that is consistent with national energy and climate policies and with long-term strategies in line with European targets.

### Quantification and statistical analyses

We perform all our quantification and statistical analyses using Python software (see key resources table), except for the binomial tests (Table S2) for which we use Excel (see key resources table). We report key statistical information to each analysis in the captions and legends of our figures and tables and describe specific calculation methods in the method details section. Table S2 presents results of binomial tests with statistical significance values (∗∗∗*p* ≤ 0.001, ∗∗*p* ≤ 0.01, ∗*p* ≤ 0.05).
