# Supplementary material for: Probabilities of reaching required diffusion of granular energy technologies in European countries
Source: iScience. 2025 Jan 16;28(2):111825. doi: 10.1016/j.isci.2025.111825 (PMC11848487; doi:10.1016/j.isci.2025.111825)
Supplement: Document S1. Figures S1–S31, Tables S1 and S2 and supplemental methods [file mmc1.pdf]

iScience, Volume *28*

Supplemental information

Probabilities of reaching required diffusion of granular energy technologies in European countries

Nik Zielonka and Evelina Trutnevyte

Supplemental information

Supplemental items

- Supplemental figures
  - Scores and weights of model variants
  - Validation of final probabilistic projections
  - Probabilistic projections
  - Maps – Distribution of capacities across Europe
  - Maps – Probability of countries to reach required quantities
  - Maps – Probability of countries to reach targets of national implementation reports
- Supplemental tables

Supplemental methods

- S1 Model variants to create probabilistic projections
- S2 S-curves
- S3 Data on maximum potential capacities

Supplemental references

Supplemental items

Supplemental figures

Scores and weights of model variants

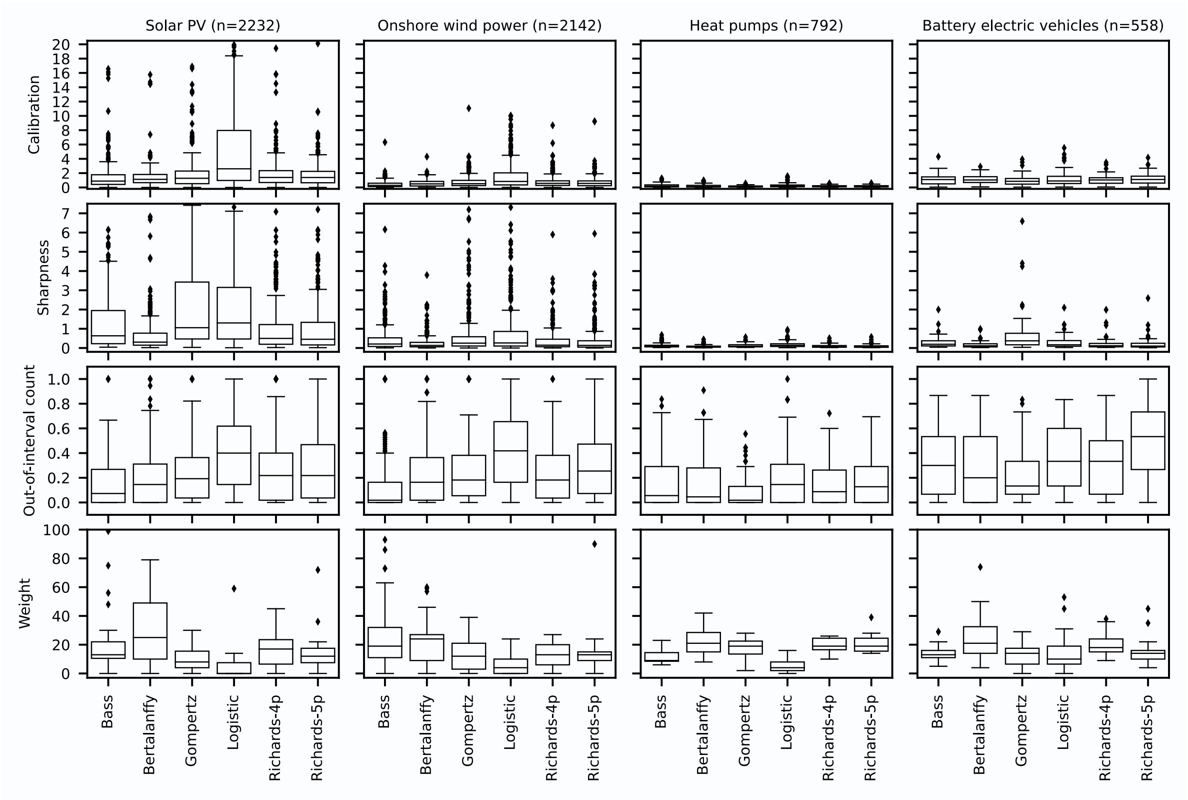

Figure S1. Box plots showing the distribution of penalties in terms of calibration (accuracy of projections), sharpness (width of projection intervals), and out-of-interval count (of historically observed data points), as well as the weights that the projections of S-curve models receive in hindcasting for the final probabilistic projections of solar photovoltaics (PV), onshore wind power, heat pumps, and battery electric vehicles across all countries. Steps 3-4 in the Method details (STAR methods) in the main article describe the hindcasting procedure for calculating the penalties and weights. The box plots show the median, the first and third quartiles, while whiskers show the range of values excluding outliers. Outliers can lay outside the shown plots. The lower the penalties, the better the performance of the projections. The resulting weights based on the inverse of the squared weighted sum of the three penalties of calibration, sharpness, and out-of-interval count are sums over all model variants that use the indicated S-curve (each variant is a combination of six S-curve models, three curve fitting variants, and four historical interval lengths). The penalties of calibration, sharpness, and out-of-interval count are means over all hindcasting years, i.e., 10 for electricity generation technologies and heat pumps, and 5 for passenger vehicles. The sample size n for each technology's box plots is the number of countries with projections for the technology (maximum 39) times the number of model variants (maximum 72 per country). See Figure S2 for offshore wind power, biogases, hybrid vehicles, and fuel cell electric vehicles. See Figure S3 and Figure S4 for box plots of the penalties and weights grouped by curve fitting variants and Figure S5 and Figure S6 grouped by historical interval lengths.

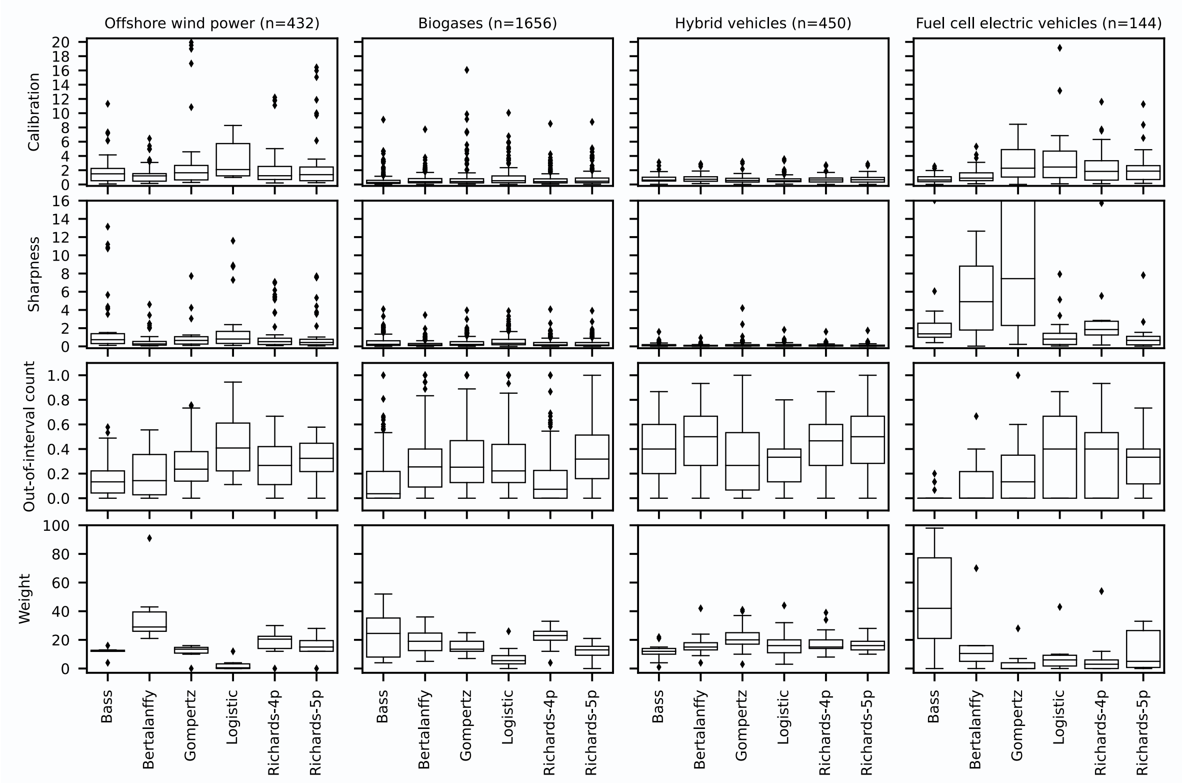

Figure S2. Box plots showing the distribution of penalties in terms of calibration (accuracy of projections), sharpness (width of projection intervals), and out-of-interval count (of historically observed data points), as well as the weights that the projections of S-curve models receive in hindcasting for the final probabilistic projections of offshore wind power, biogases, hybrid vehicles, and fuel cell electric vehicles across all countries. Steps 3-4 in the Method details (STAR methods) in the main article describe the hindcasting procedure for calculating the penalties and weights. The box plots show the median, the first and third quartiles, while whiskers show the range of values excluding outliers. Outliers can lay outside the shown plots. The lower the penalties, the better the performance of the projections. The resulting weights based on the inverse of the squared weighted sum of the three penalties of calibration, sharpness, and out-of-interval count are sums over all model variants that use the indicated S-curve (each variant is a combination of six S-curve models, three curve fitting variants, and four numbers of historical data years). The penalties of calibration, sharpness, and out-of-interval count are means over all hindcasting years, i.e., 10 for electricity generation technologies and heat pumps, and 5 for passenger vehicles. The sample size n for each technology's box plots is the number of countries with projections for the technology (maximum 39) times the number of model variants (maximum 72 per country). See Figure S1 for solar photovoltaics, onshore wind power, heat pumps, and battery electric vehicles. See Figure S3 and Figure S4 for box plots of the penalties and weights grouped by curve fitting variants and Figure S5 and Figure S6 grouped by historical interval lengths.

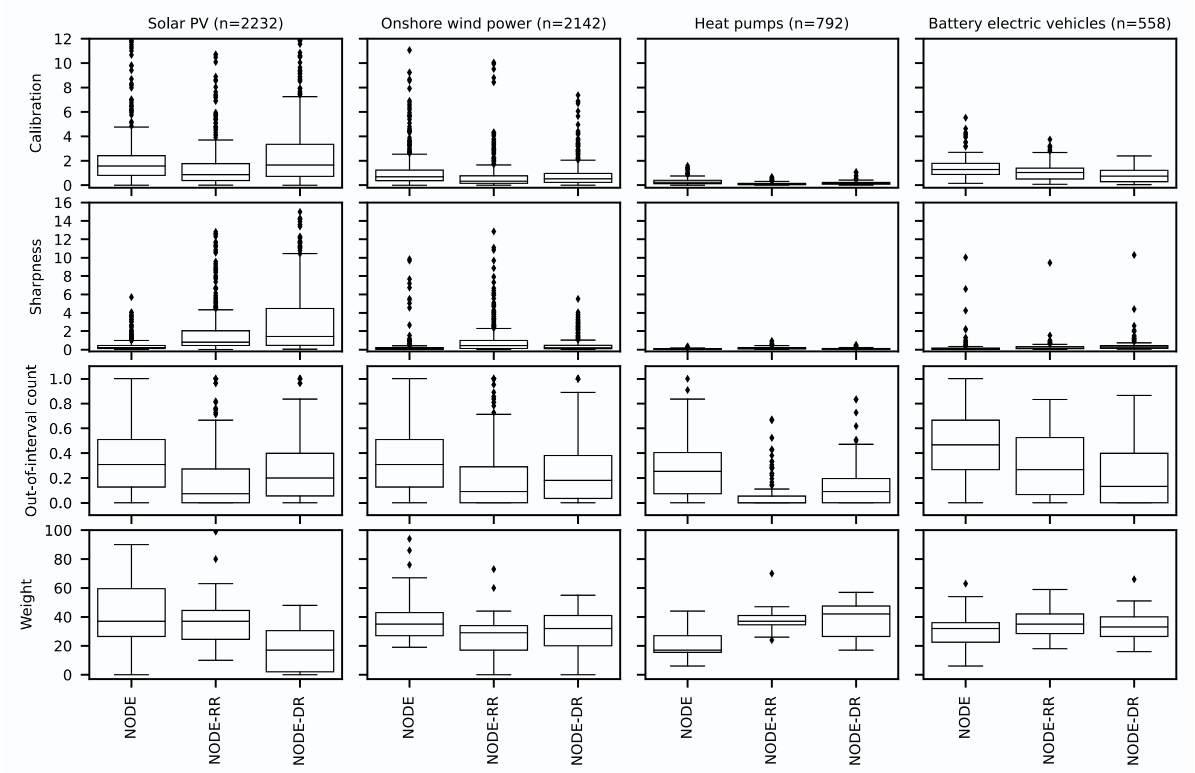

Figure S3. Box plots showing the distribution of penalties in terms of calibration (accuracy of projections), sharpness (width of projection intervals), and out-of-interval count (of historically observed data points), as well as the weights that the projections of curve fitting variants receive in hindcasting for the final projections of solar photovoltaics (PV), onshore wind power, heat pumps, and battery electric vehicles across all countries. The curve fitting variants are Near-Optimal Differential Evolution (NODE), NODE with Recent Rates (NODE-RR), and NODE with Distribution of Rates (DR). Steps 3-4 in the Method details (STAR methods) in the main article describe the hindcasting procedure for calculating the penalties and weights. The box plots show the median, the first and third quartiles, while whiskers show the range of values excluding outliers. Outliers can lay outside the shown plots. The lower the penalties, the better the performance of the projections. The resulting weights based on the inverse of the squared weighted sum of the three penalties of calibration, sharpness, and out-of-interval count are sums over all model variants that use the indicated curve fitting variant (each variant is a combination of six S-curve models, three curve fitting variants, and four numbers of historical data years). The penalties of calibration, sharpness, and out-of-interval count are means over all hindcasting years, i.e., 10 for electricity generation technologies and heat pumps, and 5 for passenger vehicles. The sample size n for each technology's box plots is the number of countries with projections for the technology (maximum 39) times the number of model variants (maximum 72 per country). See Figure S4 for offshore wind power, biogases, hybrid vehicles, and fuel cell electric vehicles. See Figure S1 and Figure S2 for box plots of the penalties and weights grouped by S-curve models and Figure S5 and Figure S6 grouped by historical interval lengths.

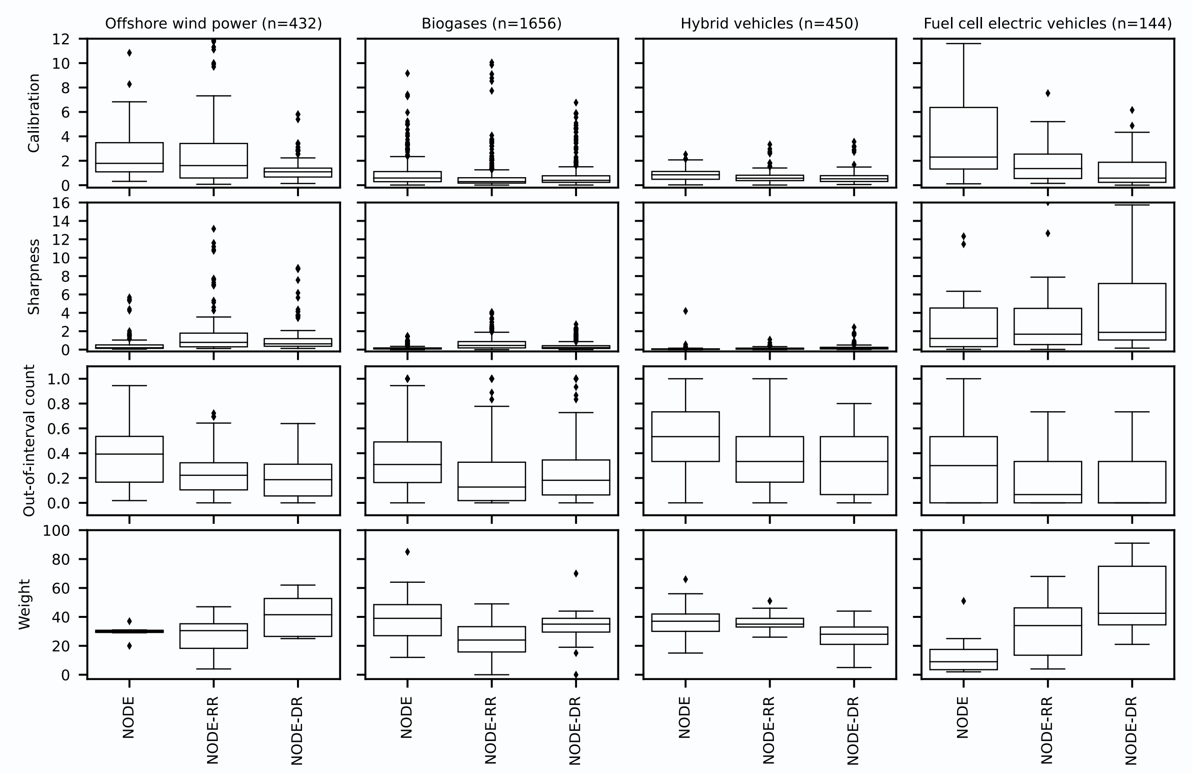

Figure S4. Box plots showing the distribution of penalties in terms of calibration (accuracy of projections), sharpness (width of projection intervals), and out-of-interval count (of historically observed data points), as well as the weights that the projections of curve fitting variants receive in hindcasting for the final projections of offshore wind power, biogases, hybrid vehicles, and fuel cell electric vehicles across all countries. The curve fitting variants are Near-Optimal Differential Evolution (NODE), NODE with Recent Rates (NODE-RR), and NODE with Distribution of Rates (DR). Steps 3-4 in the Method details (STAR methods) in the main article describe the hindcasting procedure for calculating the penalties and weights. The box plots show the median, the first and third quartiles, while whiskers show the range of values excluding outliers. Outliers can lay outside the shown plots. The lower the penalties, the better the performance of the projections. The resulting weights based on the inverse of the squared weighted sum of the three penalties of calibration, sharpness, and out-of-interval count are sums over all model variants that use the indicated curve fitting variant (each variant is a combination of six S-curve models, three curve fitting variants, and four numbers of historical data years). The penalties of calibration, sharpness, and out-of-interval count are means over all hindcasting years, i.e., 10 for electricity generation technologies and heat pumps, and 5 for passenger vehicles. The sample size n for each technology's box plots is the number of countries with projections for the technology (maximum 39) times the number of model variants (maximum 72 per country). See Figure S3 for solar photovoltaics, onshore wind power, heat pumps, and battery electric vehicles. See Figure S1 and Figure S2 for box plots of the penalties and weights grouped by S-curve models and Figure S5 and Figure S6 grouped by historical interval lengths.

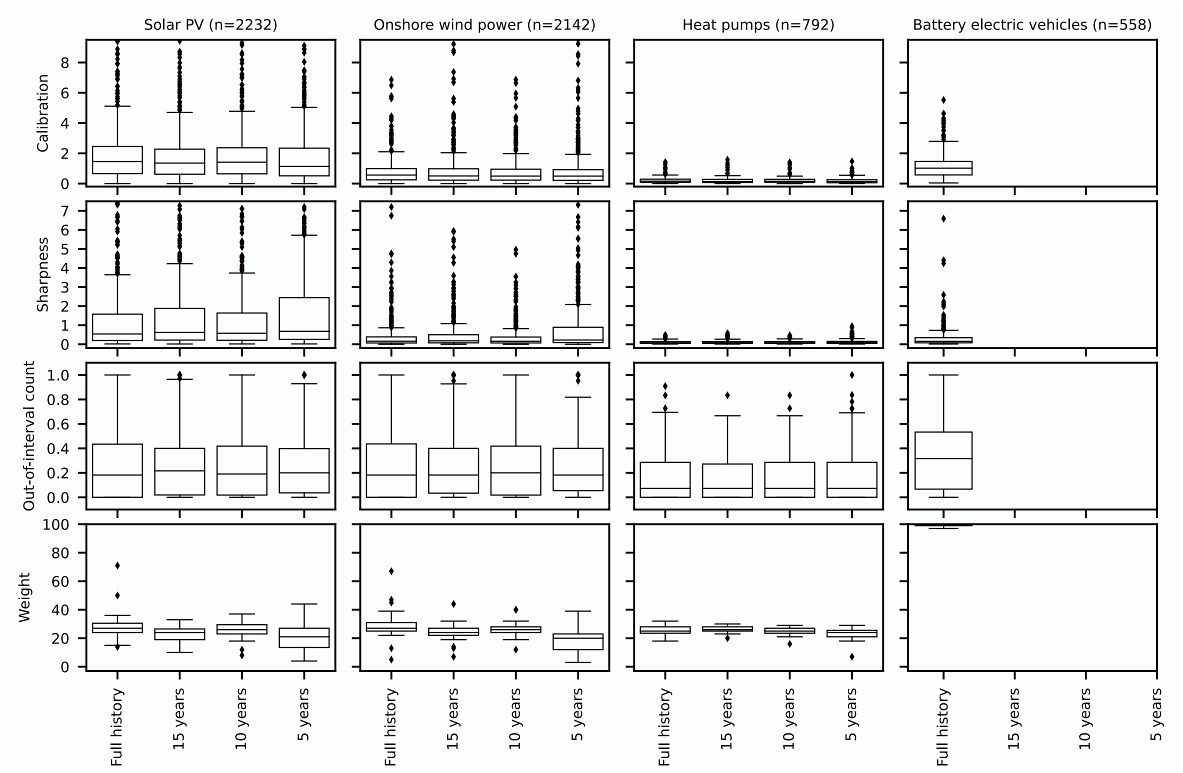

Figure S5. Box plots showing the distribution of penalties in terms of calibration (accuracy of projections), sharpness (width of projection intervals), and out-of-interval count (of historically observed data points), as well as the weights that the projections with different historical interval lengths receive in hindcasting for the final probabilistic projections of solar photovoltaics (PV), onshore wind power, heat pumps, and battery electric vehicles across all countries. Steps 3-4 in the Method details (STAR methods) in the main article describe the hindcasting procedure for calculating the penalties and weights. The box plots show the median, the first and third quartiles, while whiskers show the range of values excluding outliers. Outliers can lay outside the shown plots. The lower the penalties, the better the performance of the projections. The resulting weights based on the inverse of the squared weighted sum of the three penalties of calibration, sharpness, and out-of-interval count are sums over all model variants that use the indicated historical interval length (each variant is a combination of six S-curve models, three curve fitting variants, and four numbers of historical data years). As there is no limited historical interval length for electric vehicles, the weight for the full history variant is always 100%. The penalties of calibration, sharpness, and out-of-interval count are means over all hindcasting years, i.e., 10 for electricity generation technologies and heat pumps, and 5 for passenger vehicles. The sample size n for each technology's box plots is the number of countries with projections for the technology (maximum 39) times the number of model variants (maximum 72 per country). See Figure S6 for offshore wind power, biogases, hybrid vehicles, and fuel cell electric vehicles. See Figure S1 and Figure S2 for box plots of the penalties and weights grouped by S-curve models and Figure S3 and Figure S4 grouped by curve fitting variants.

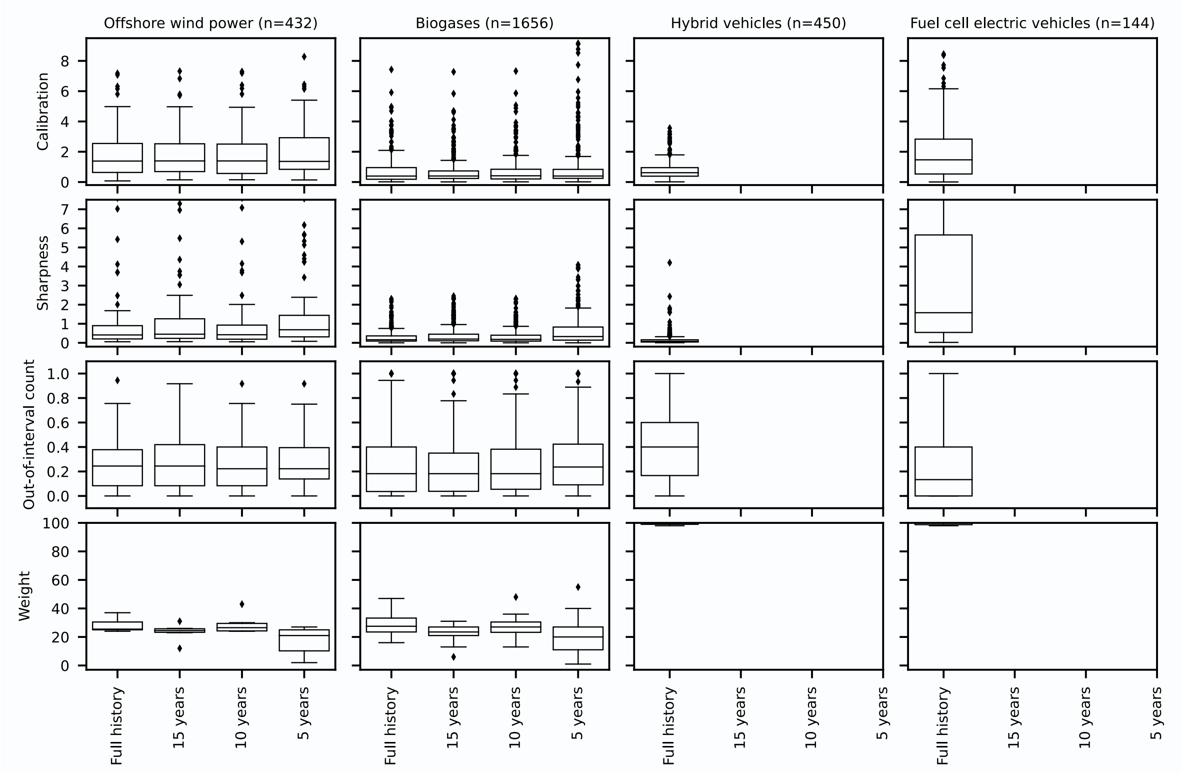

Figure S6. Box plots showing the distribution of penalties in terms of calibration (accuracy of projections), sharpness (width of projection intervals), and out-of-interval count (of historically observed data points), as well as the weights that the projections with different historical interval lengths receive in hindcasting for the final probabilistic projections of offshore wind power, biogases, hybrid vehicles, and fuel cell electric vehicles across all countries. Steps 3-4 in the Method details (STAR methods) in the main article describe the hindcasting procedure for calculating the penalties and weights. The box plots show the median, the first and third quartiles, while whiskers show the range of values excluding outliers. Outliers can lay outside the shown plots. The lower the penalties, the better the performance of the projections. The resulting weights based on the inverse of the squared weighted sum of the three penalties of calibration, sharpness, and out-of-interval count are sums over all model variants that use the indicated historical interval length (each variant is a combination of six S-curve models, three curve fitting variants, and four numbers of historical data years). As there is no limited historical interval length for electric vehicles, the weight for the full history variant is always 100%. The penalties of calibration, sharpness, and out-of-interval count are means over all hindcasting years, i.e., 10 for electricity generation technologies and heat pumps, and 5 for passenger vehicles. The sample size n for each technology's box plots is the number of countries with projections for the technology (maximum 39) times the number of model variants (maximum 72 per country). See Figure S5 for solar photovoltaics, onshore wind power, heat pumps, and battery electric vehicles. See Figure S1 and Figure S2 for box plots of the penalties and weights grouped by S-curve models and Figure S3 and Figure S4 grouped by curve fitting variants.

Validation of final probabilistic projections

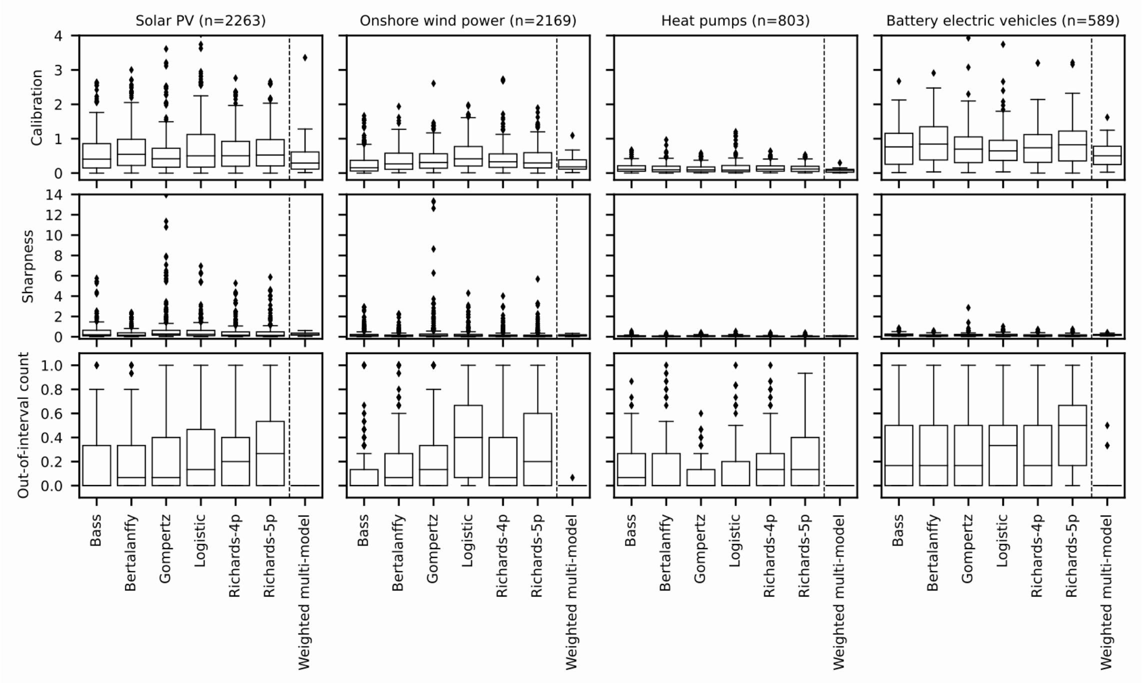

Figure S7. Box plots showing the distribution of penalties in terms of calibration (accuracy of projections), sharpness (width of projection intervals), and out-of-interval count (of historically observed data points) that the projections of S-curve models receive in hindcasting from 2018-2022 compared to the final probabilistic projections of the weighted multi-model of solar photovoltaics (PV), onshore wind power, heat pumps, and battery electric vehicles across all countries. Steps 3-4 in the Method details (STAR methods) in the main article describe the hindcasting procedure for calculating the penalties. The box plots show the median, the first and third quartiles, while whiskers show the range of values excluding outliers. Outliers can lay outside the shown plots. The lower the penalties, the better the performance of the projections. The penalties of calibration, sharpness, and out-of-interval count are means over all hindcasting years, i.e., the last 5 for electricity generation technologies and heat pumps, and the last 3 for passenger vehicles. The sample size n for each technology's box plots is the number of countries with projections for the technology (maximum 39) times the number of model variants (maximum 72 per country). See Figure S8 for offshore wind power, biogases, hybrid vehicles, and fuel cell electric vehicles. See Figure S9 and Figure S10 for box plots of the penalties and weights grouped by curve fitting variants and Figure S11 and Figure S12 grouped by historical interval lengths.

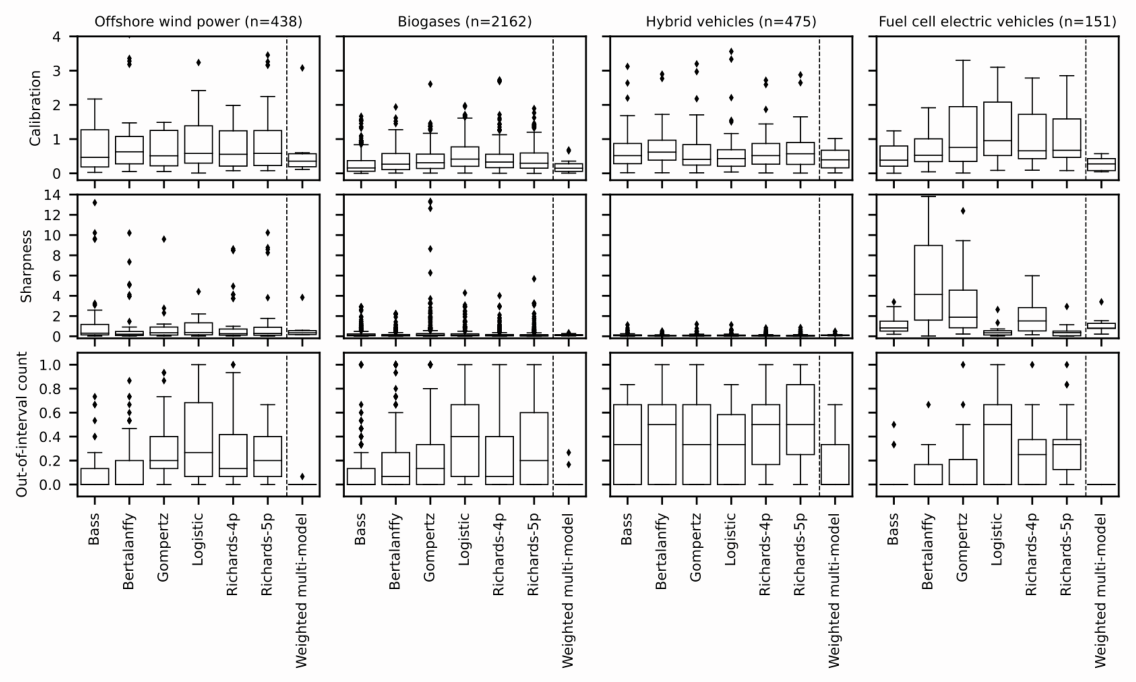

Figure S8. Box plots showing the distribution of penalties in terms of calibration (accuracy of projections), sharpness (width of projection intervals), and out-of-interval count (of historically observed data points) that the projections of S-curve models receive in hindcasting from 2018-2022 compared to the final probabilistic projections of the weighted multi-model of offshore wind power, biogases, hybrid vehicles, and fuel cell electric vehicles across all countries. Steps 3-4 in the Method details (STAR methods) in the main article describe the hindcasting procedure for calculating the penalties. The box plots show the median, the first and third quartiles, while whiskers show the range of values excluding outliers. Outliers can lay outside the shown plots. The lower the penalties, the better the performance of the projections. The penalties of calibration, sharpness, and out-of-interval count are means over all hindcasting years, i.e., the last 5 for electricity generation technologies and heat pumps, and the last 3 for passenger vehicles. The sample size n for each technology's box plots is the number of countries with projections for the technology (maximum 39) times the number of model variants (maximum 72 per country). See Figure S7 for solar photovoltaics, onshore wind power, heat pumps, and battery electric vehicles. See Figure S9 and Figure S10 for box plots of the penalties and weights grouped by curve fitting variants and Figure S11 and Figure S12 grouped by historical interval lengths.

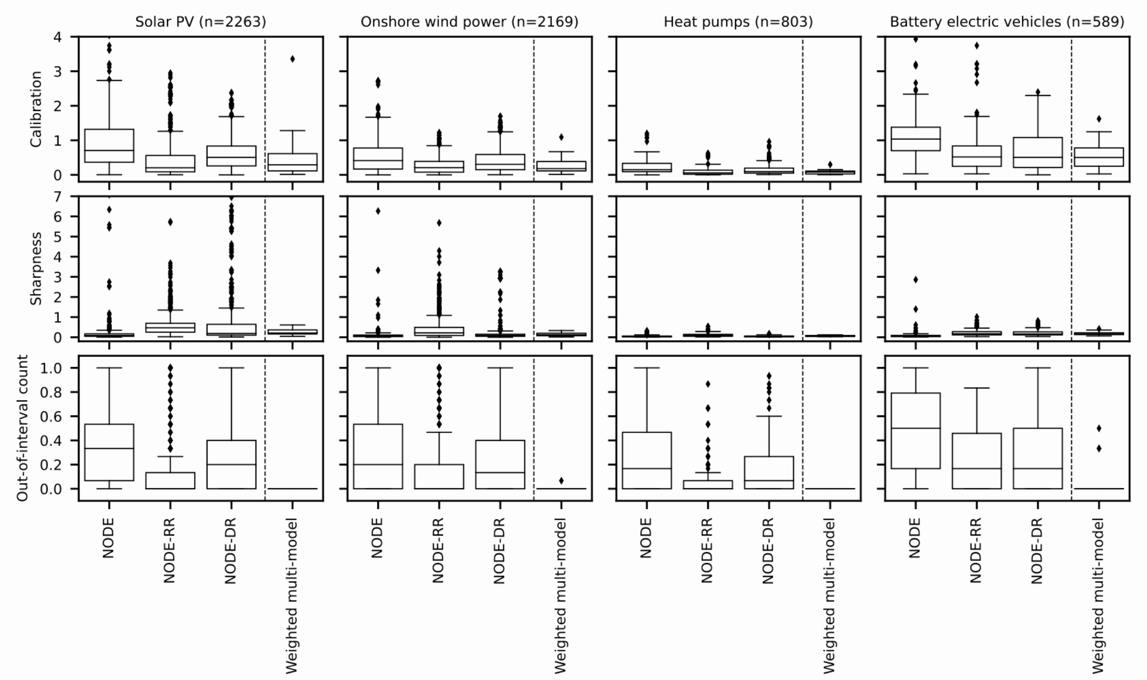

Figure S9. Box plots showing the distribution of penalties in terms of calibration (accuracy of projections), sharpness (width of projection intervals), and out-of-interval count (of historically observed data points) that the projections of curve fitting variants receive in hindcasting from 2018-2022 compared to the final probabilistic projections of the weighted multi-model of solar photovoltaics (PV), onshore wind power, heat pumps, and battery electric vehicles across all countries. The curve fitting variants are Near-Optimal Differential Evolution (NODE), NODE with Recent Rates (NODE-RR), and NODE with Distribution of Rates (DR). Steps 3-4 in the Method details (STAR methods) in the main article describe the hindcasting procedure for calculating the penalties. The box plots show the median, the first and third quartiles, while whiskers show the range of values excluding outliers. Outliers can lay outside the shown plots. The lower the penalties, the better the performance of the projections. The penalties of calibration, sharpness, and out-of-interval count are means over all hindcasting years, i.e., the last 5 for electricity generation technologies and heat pumps, and the last 3 for passenger vehicles. The sample size n for each technology's box plots is the number of countries with projections for the technology (maximum 39) times the number of model variants (maximum 72 per country). See Figure S10 for offshore wind power, biogases, hybrid vehicles, and fuel cell electric vehicles. See Figure S7 and Figure S8 for box plots of the penalties and weights grouped by S-curve models and Figure S11 and Figure S12 grouped by numbers of historical interval lengths.

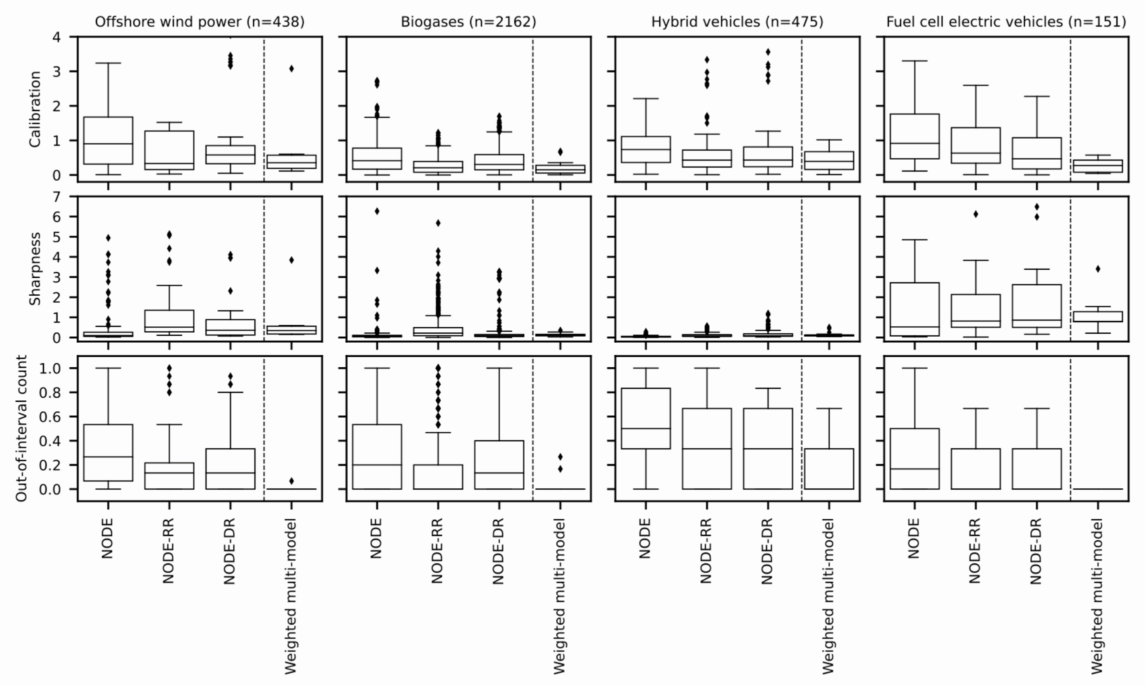

Figure S10. Box plots showing the distribution of penalties in terms of calibration (accuracy of projections), sharpness (width of projection intervals), and out-of-interval count (of historically observed data points) that the projections of curve fitting variants receive in hindcasting from 2018-2022 compared to the final probabilistic projections of the weighted multi-model of offshore wind power, biogases, hybrid vehicles, and fuel cell electric vehicles across all countries. The curve fitting variants are Near-Optimal Differential Evolution (NODE), NODE with Recent Rates (NODE-RR), and NODE with Distribution of Rates (DR). Steps 3-4 in the Method details (STAR methods) in the main article describe the hindcasting procedure for calculating the penalties. The box plots show the median, the first and third quartiles, while whiskers show the range of values excluding outliers. Outliers can lay outside the shown plots. The lower the penalties, the better the performance of the projections. The penalties of calibration, sharpness, and out-of-interval count are means over all hindcasting years, i.e., the last 5 for electricity generation technologies and heat pumps, and the last 3 for passenger vehicles. The sample size n for each technology's box plots is the number of countries with projections for the technology (maximum 39) times the number of model variants (maximum 72 per country). See Figure S9 for solar photovoltaics, onshore wind power, heat pumps, and battery electric vehicles. See Figure S7 and Figure S8 for box plots of the penalties and weights grouped by S-curve models and Figure S11 and Figure S12 grouped by numbers of historical interval lengths.

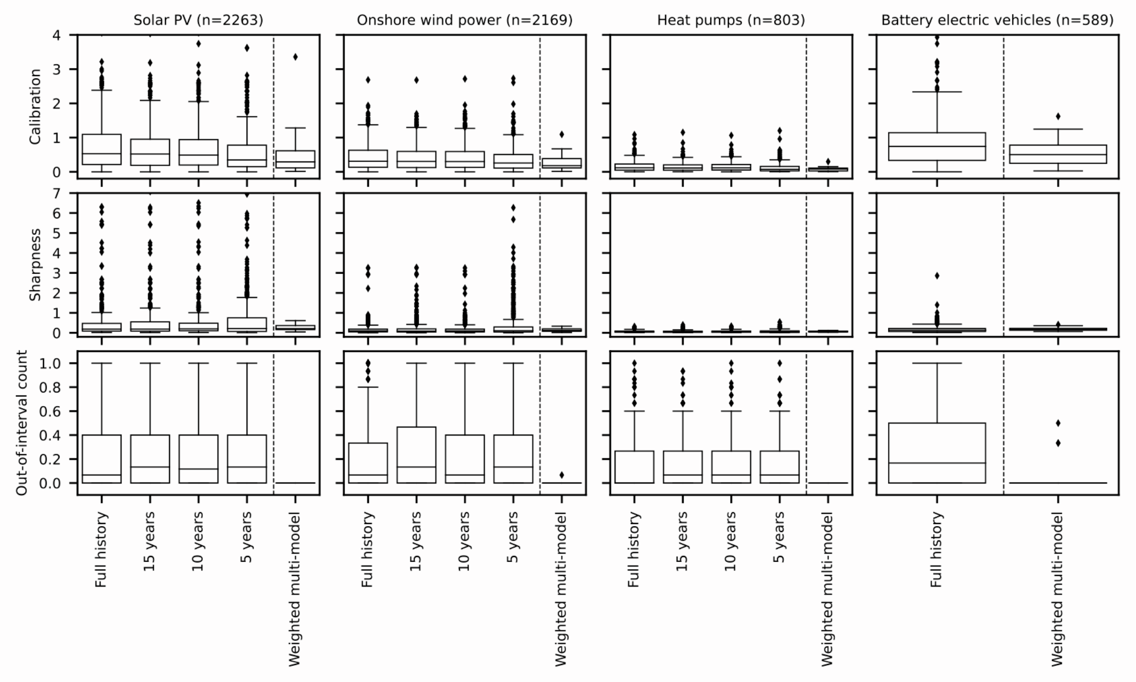

Figure S11. Box plots showing the distribution of penalties in terms of calibration (accuracy of projections), sharpness (width of projection intervals), and out-of-interval count (of historically observed data points) that the projections with different historical interval lengths receive in hindcasting from 2018-2022 compared to the final probabilistic projections of the weighted multi-model of solar photovoltaics (PV), onshore wind power, heat pumps, and battery electric vehicles across all countries. Steps 3-4 in the Method details (STAR methods) in the main article describe the hindcasting procedure for calculating the penalties. The box plots show the median, the first and third quartiles, while whiskers show the range of values excluding outliers. Outliers can lay outside the shown plots. The lower the penalties, the better the performance of the projections. As there is no limited historical interval length for electric vehicles, the weight for the full history variant is always 100%. The penalties of calibration, sharpness, and out-of-interval count are means over all hindcasting years, i.e., the last 5 for electricity generation technologies and heat pumps, and the last 3 for passenger vehicles. The sample size n for each technology's box plots is the number of countries with projections for the technology (maximum 39) times the number of model variants (maximum 72 per country). See Figure S12 for offshore wind power, biogases, hybrid vehicles, and fuel cell electric vehicles. See Figure S7 and Figure S8 for box plots of the penalties and weights grouped by S-curve models and Figure S9 and Figure S10 grouped by curve fitting variants.

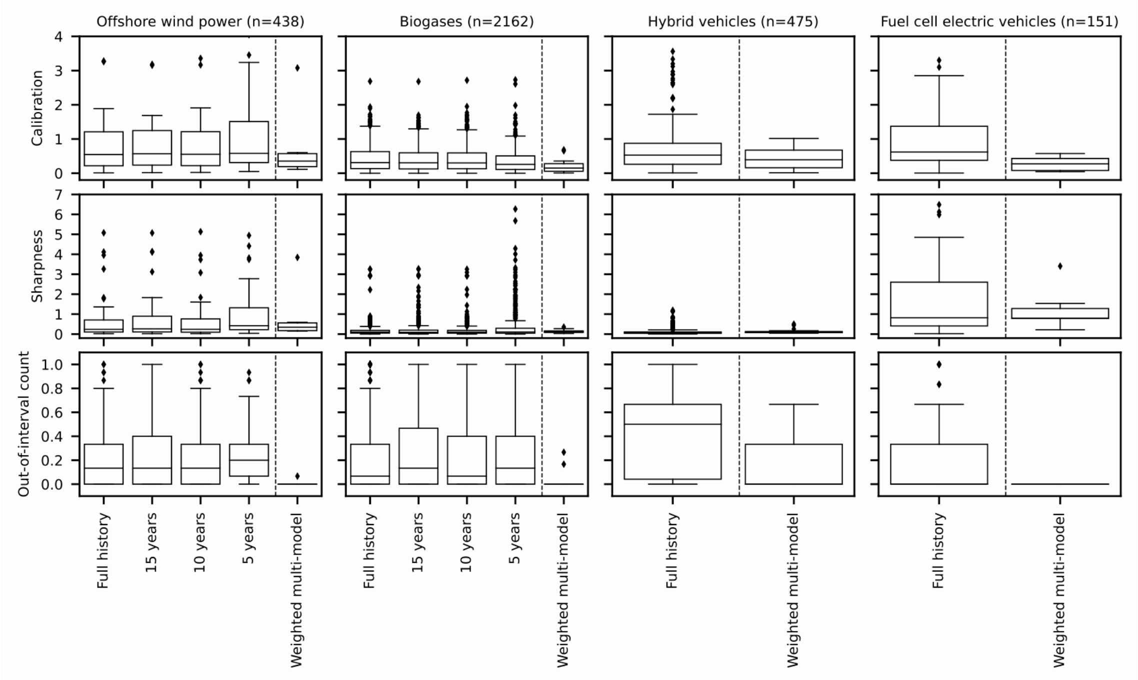

Figure S12. Box plots showing the distribution of penalties in terms of calibration (accuracy of projections), sharpness (width of projection intervals), and out-of-interval count (of historically observed data points) that the projections with different historical interval lengths receive in hindcasting from 2018-2022 compared to the final probabilistic projections of the weighted multi-model of offshore wind power, biogases, hybrid vehicles, and fuel cell electric vehicles across all countries. Steps 3-4 in the Method details (STAR methods) in the main article describe the hindcasting procedure for calculating the penalties. The box plots show the median, the first and third quartiles, while whiskers show the range of values excluding outliers. Outliers can lay outside the shown plots. The lower the penalties, the better the performance of the projections. As there is no limited historical interval length for electric vehicles, the weight for the full history variant is always 100%. The penalties of calibration, sharpness, and out-of-interval count are means over all hindcasting years, i.e., the last 5 for electricity generation technologies and heat pumps, and the last 3 for passenger vehicles. The sample size n for each technology's box plots is the number of countries with projections for the technology (maximum 39) times the number of model variants (maximum 72 per country). See Figure S11 for solar photovoltaics, onshore wind power, heat pumps, and battery electric vehicles. See Figure S7 and Figure S8 for box plots of the penalties and weights grouped by S-curve models and Figure S9 and Figure S10 grouped by curve fitting variants.

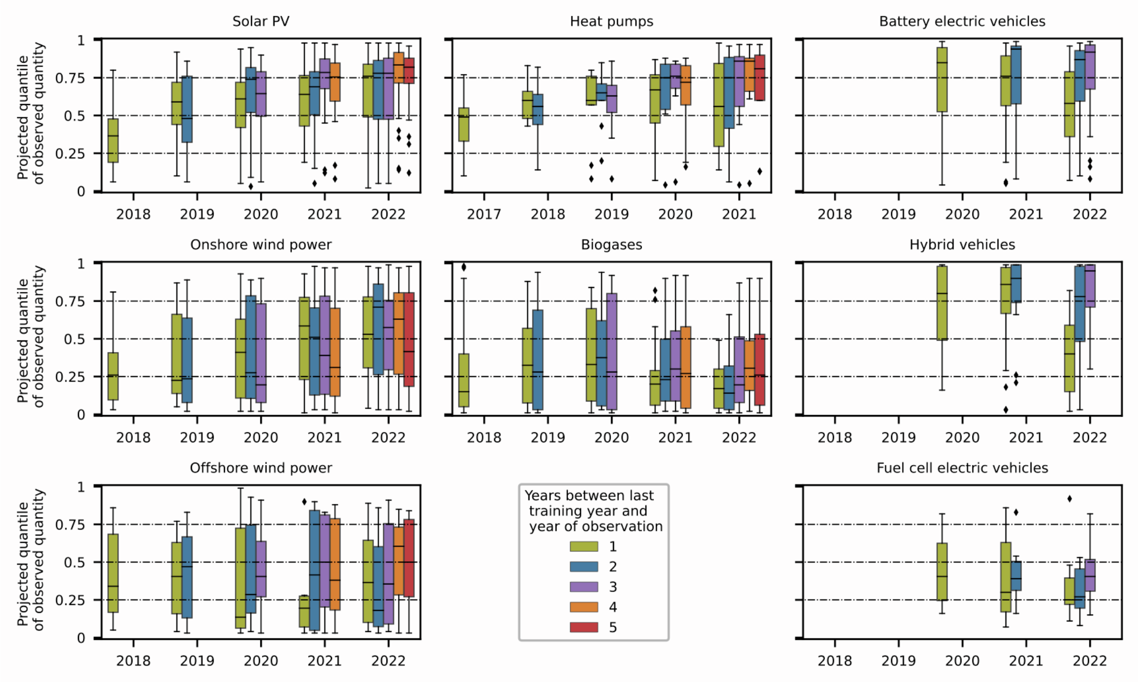

Figure S13. Box plots showing which quantile the probabilistic projections estimated for out-of-sample capacities that are observed for a technology between 2017-2022, identified in out-of-sample testing. The testing follows the same logic that steps 3-4 in the Method details (STAR methods) in the main article describe. Each box plot represents the distribution of quantiles across all countries with a projection for the particular technology. The box plots are grouped by the year of observation and separated by how many years lay between the last year of training and the year of observation, thereby representing 1-year-ahead to 5-year-ahead projections. The black dash-dotted lines indicate the median and the interquartile range of the quantiles. The box plots show the median, the first and third quartiles, while whiskers show the range of values excluding outliers. Outliers can lay outside the shown plots.

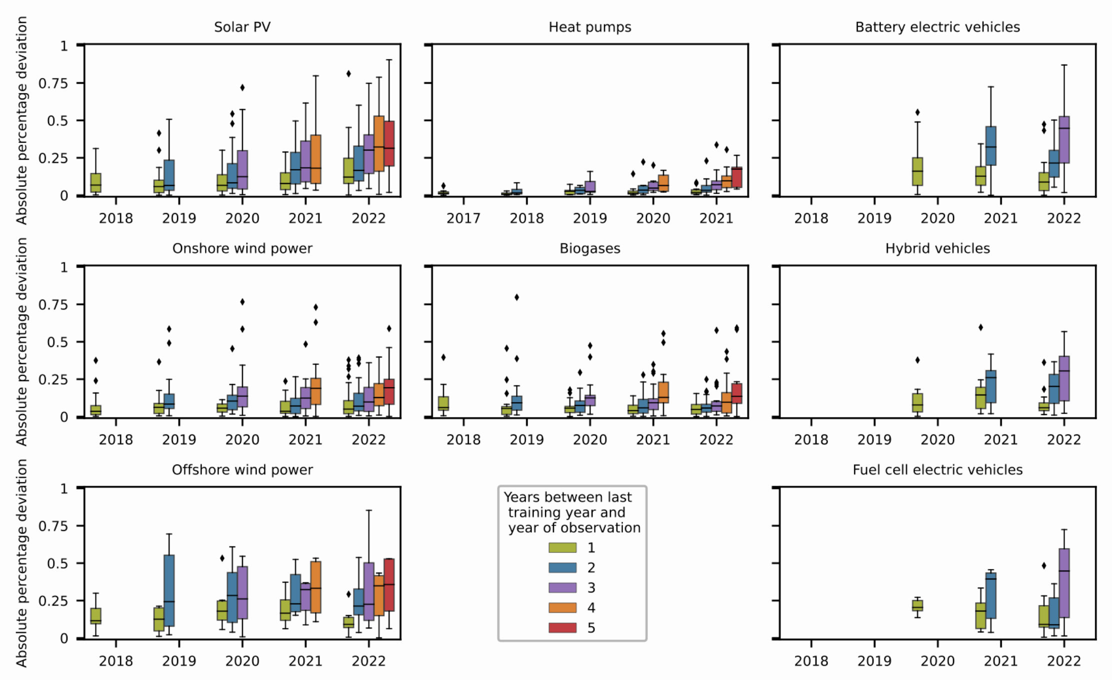

Figure S14. Box plots showing the absolute percentage deviation between the projected median and out-of-sample capacities that are observed for a technology between 2017-2022, identified in out-of-sample testing. The testing follows the same logic that steps 3-4 in the Method details (STAR methods) in the main article describe. Each box plot represents the distribution of errors across all countries with a projection for the particular technology. The box plots are grouped by the year of observation and separated by how many years lay between the last year of training and the year of observation, thereby representing 1-year-ahead to 5-year-ahead projections. The box plots show the median, the first and third quartiles, while whiskers show the range of values excluding outliers. Outliers can lay outside the shown plots.

Probabilistic projections

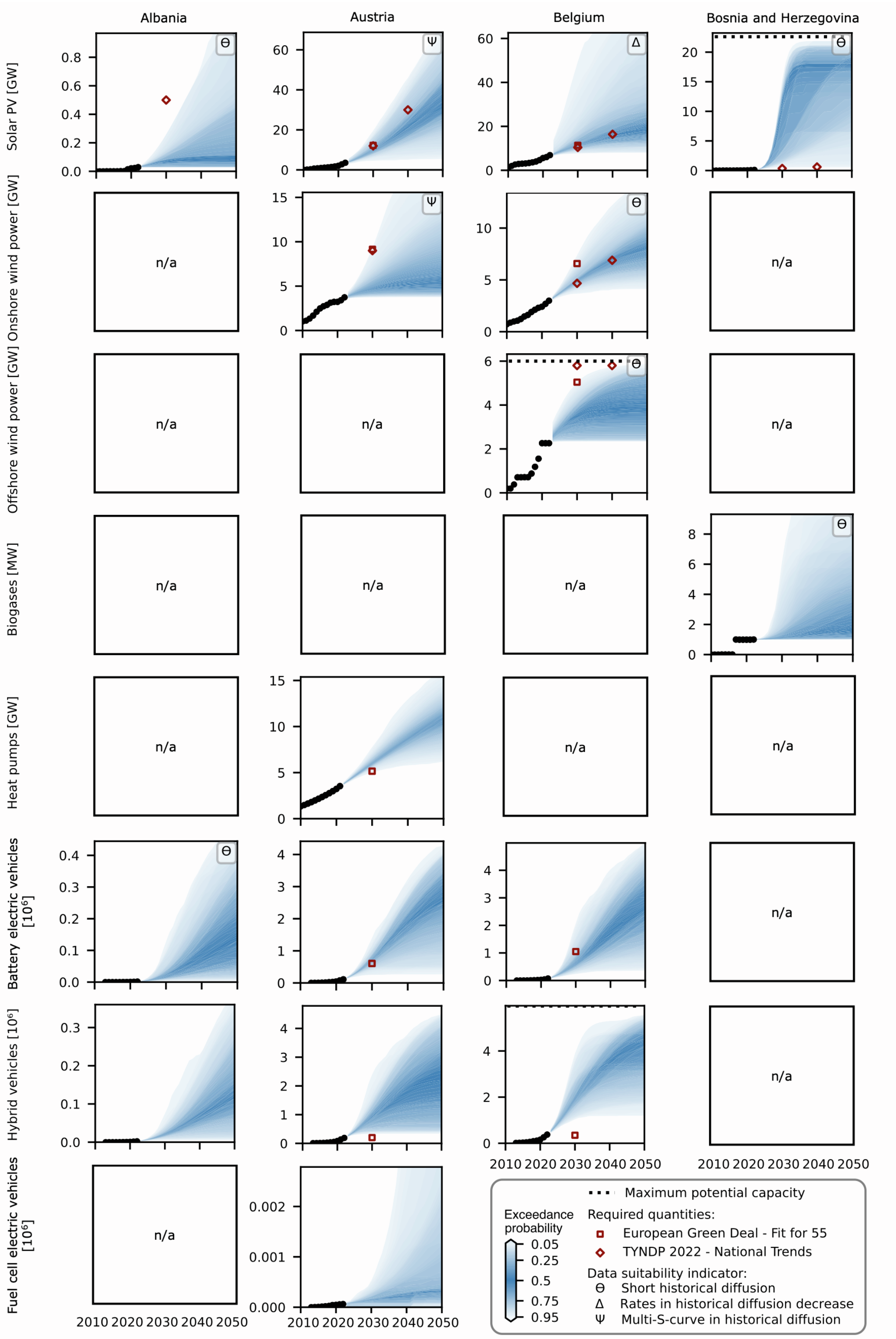

Figure S15. Probabilistic projections for all eight investigated technologies in Albania, Austria, Belgium, and Bosnia and Herzegovina, including training data until 2022, respectively 2021 for heat pumps. Training data starts in 1990, respectively 2013 for electric vehicles. The probabilistic density intervals show the probability that a quantity will be reached or exceeded if the diffusion follows current trends. The color gradient describes the quantiles of the projected capacities: the darker the color, the closer the capacity is to the median. The black dots show historical capacities and the black dotted lines, if visible, the maximum potential capacity that a country can install (see STAR methods in the main article). The red squares and diamonds show required quantities for the energy transition, estimated in scenarios of European Commission[1,2] and Ten Year Network Development Plan[3] that are consistent with the European Green Deal policy package “Fit for 55”, and national energy and climate policies (see STAR methods). Required quantities may be invisible if they are larger than the upper limit of the vertical axes

of the projections. The Greek letters indicate a qualitative assessment on the suitability of the historical time series data for projecting probabilistic growth (see STAR methods), where a short historical diffusion (Θ) results in less reliable projections as empirical testing is comparatively short, decreasing historical diffusion rates (Δ) may result in early saturation, and a multi-S-curve pattern (Ψ) may lead to an underestimation of future growth. Figures S16-S24 provide projections for the remaining countries. Related to Figure 1.

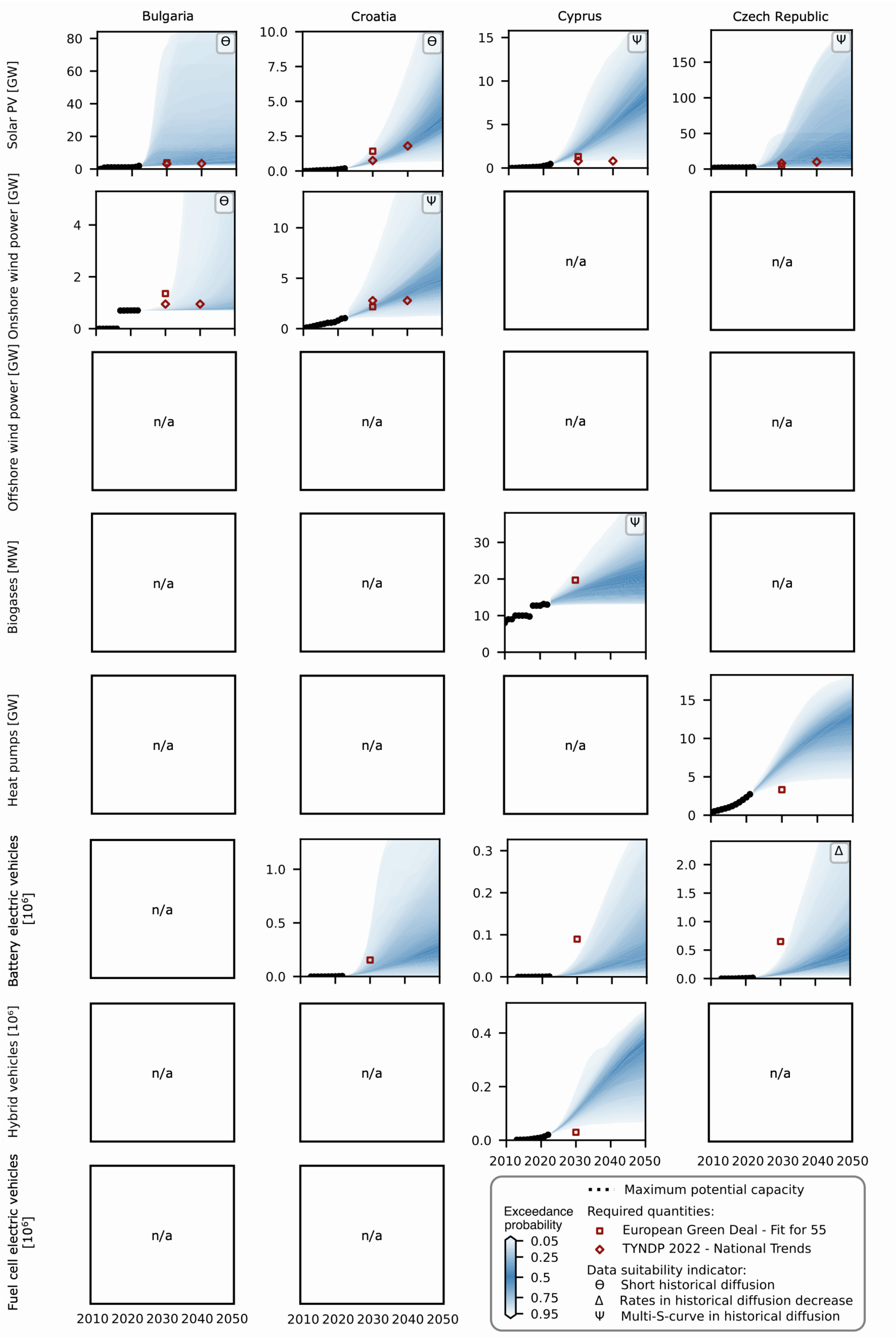

Figure S16. Probabilistic projections for all eight investigated technologies in Bulgaria, Croatia, Cyprus, and Czech Republic, including training data until 2022, respectively 2021 for heat pumps. Training data starts in 1990, respectively 2013 for electric vehicles. The probabilistic density intervals show the probability that a quantity will be reached or exceeded if the diffusion follows current trends. The color gradient describes the quantiles of the projected capacities: the darker the color, the closer the capacity is to the median. The black dots show historical capacities and the black dotted lines, if visible, the

maximum potential capacity that a country can install (see STAR methods in the main article). The red squares and diamonds show required quantities for the energy transition, estimated in scenarios of European Commission[1,2] and Ten Year Network Development Plan[3] that are consistent with the European Green Deal policy package "Fit for 55", and national energy and climate policies (see STAR methods). Required quantities may be invisible if they are larger than the upper limit of the vertical axes of the projections. The Greek letters indicate a qualitative assessment on the suitability of the historical time series data for projecting probabilistic growth (see STAR methods), where a short historical diffusion (Θ) results in less reliable projections as empirical testing is comparatively short, decreasing historical diffusion rates (Δ) may result in early saturation, and a multi-S-curve pattern (Ψ) may lead to an underestimation of future growth. Figures S15-S24 provide projections for the remaining countries. Related to Figure 1.

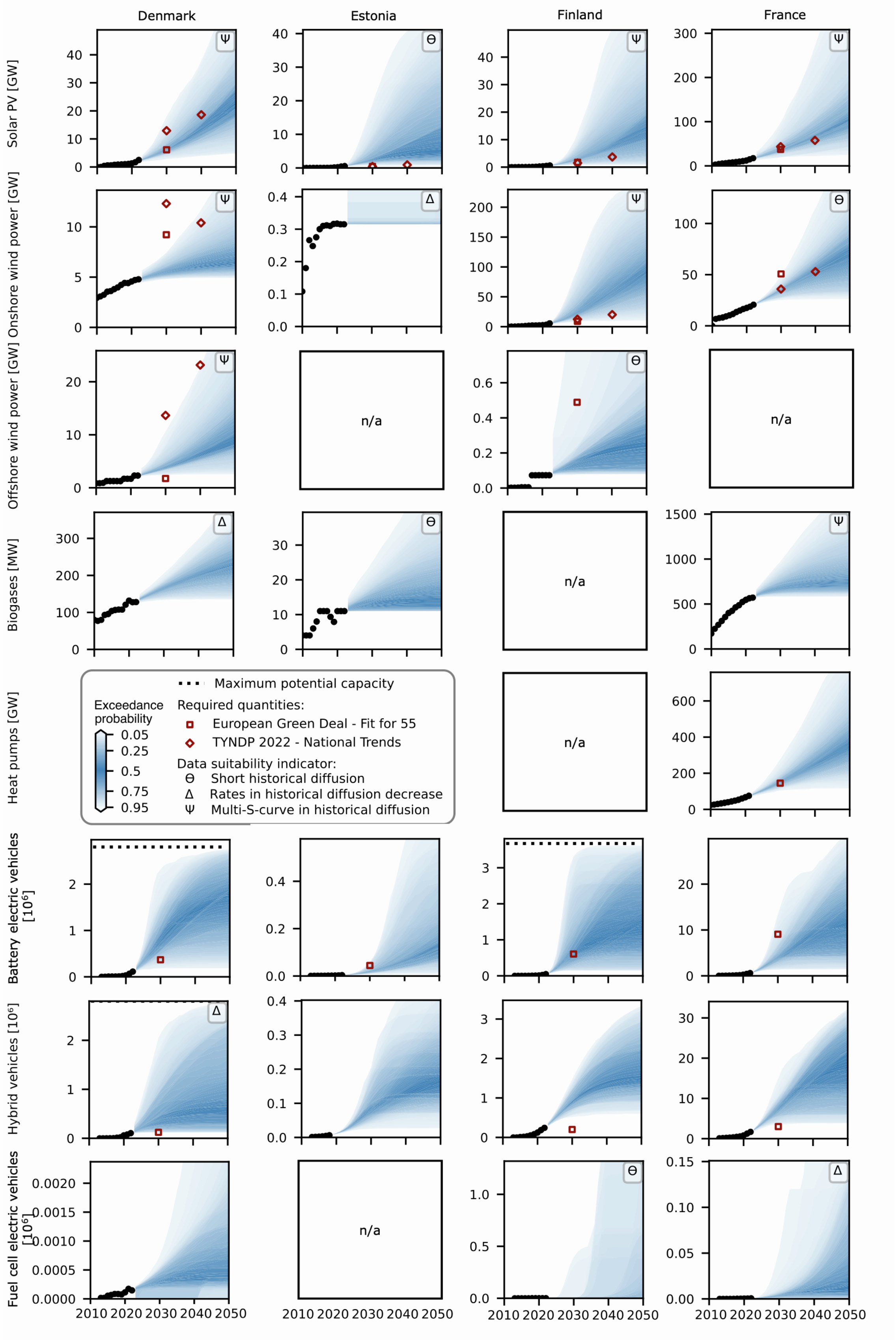

Figure S17. Probabilistic projections for all eight investigated technologies in Denmark, Estonia, Finland, and France, including training data until 2022, respectively 2021 for heat pumps. Training data starts in 1990, respectively 2013 for electric vehicles. The probabilistic density intervals show the probability that a quantity will be reached or exceeded if the diffusion follows current trends. The color gradient describes the quantiles of the projected capacities: the darker the color, the closer the capacity is to the median. The black dots show historical capacities and the black dotted lines, if visible, the maximum potential capacity that a country can install (see STAR methods in the main article). The red squares

and diamonds show required quantities for the energy transition, estimated in scenarios of European Commission[1,2] and Ten Year Network Development Plan[3] that are consistent with the European Green Deal policy package "Fit for 55", and national energy and climate policies (see STAR methods). Required quantities may be invisible if they are larger than the upper limit of the vertical axes of the projections. The Greek letters indicate a qualitative assessment on the suitability of the historical time series data for projecting probabilistic growth (see STAR methods), where a short historical diffusion (Θ) results in less reliable projections as empirical testing is comparatively short, decreasing historical diffusion rates (Δ) may result in early saturation, and a multi-S-curve pattern (Ψ) may lead to an underestimation of future growth. Figures S15-S24 provide projections for the remaining countries. Related to Figure 1.

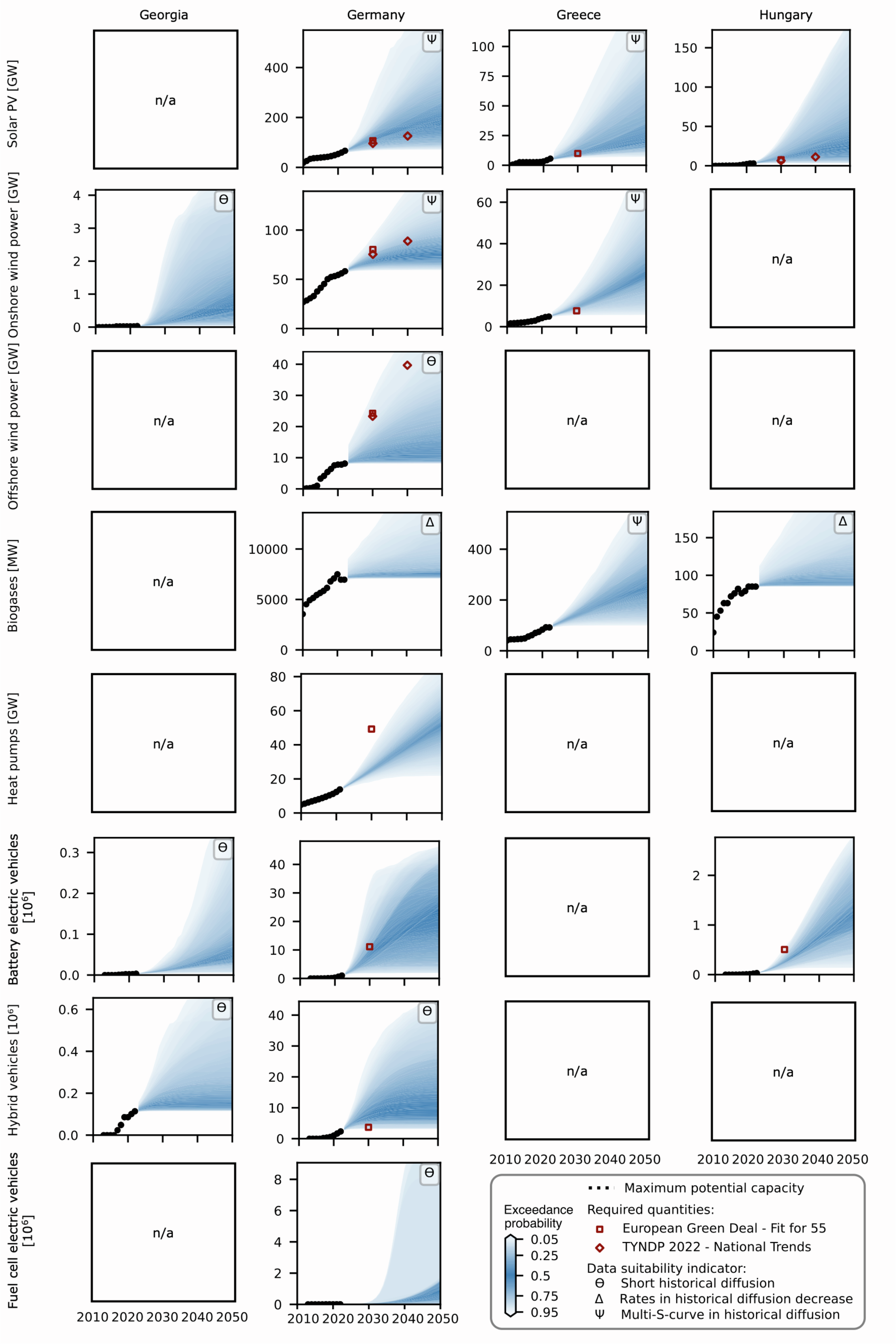

Figure S18. Probabilistic projections for all eight investigated technologies in Georgia, Germany, Greece, and Hungary, including training data until 2022, respectively 2021 for heat pumps. Training data starts in 1990, respectively 2013 for electric vehicles. The probabilistic density intervals show the probability that a quantity will be reached or exceeded if the diffusion follows current trends. The color gradient describes the quantiles of the projected capacities: the darker the color, the closer the capacity is to the median. The black dots show historical capacities and the black dotted lines, if visible, the maximum potential capacity that a country can install (see STAR methods in the main article). The red

squares and diamonds show required quantities for the energy transition, estimated in scenarios of European Commission[1,2] and Ten Year Network Development Plan[3] that are consistent with the European Green Deal policy package “Fit for 55”, and national energy and climate policies (see STAR methods). Required quantities may be invisible if they are larger than the upper limit of the vertical axes of the projections. The Greek letters indicate a qualitative assessment on the suitability of the historical time series data for projecting probabilistic growth (see STAR methods), where a short historical diffusion (Θ) results in less reliable projections as empirical testing is comparatively short, decreasing historical diffusion rates (Δ) may result in early saturation, and a multi-S-curve pattern (Ψ) may lead to an underestimation of future growth. Figures S15-S24 provide projections for the remaining countries. Related to Figure 1.

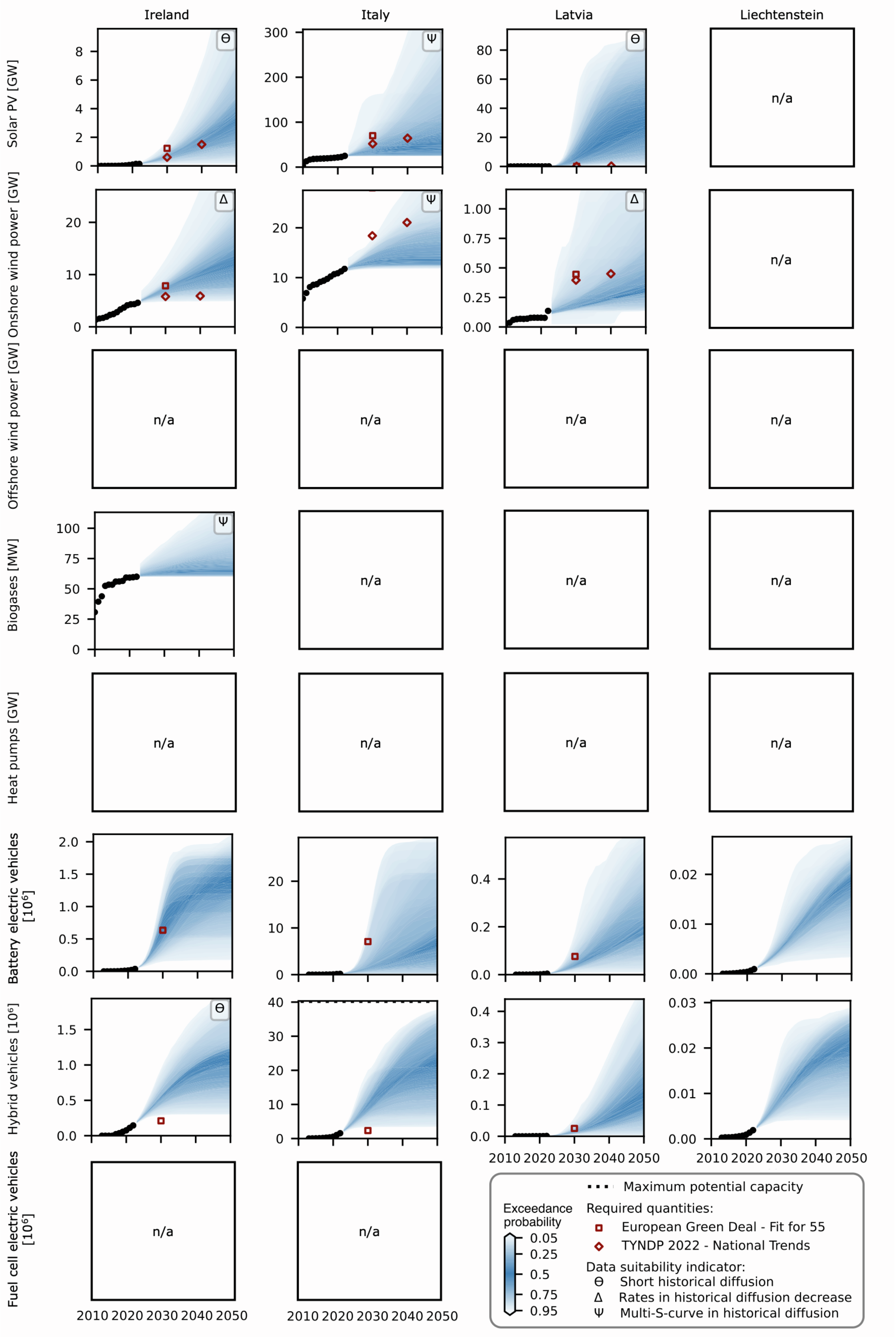

Figure S19. Probabilistic projections for all eight investigated technologies in Ireland, Italy, Latvia, and Liechtenstein, including training data until 2022, respectively 2021 for heat pumps. Training data starts in 1990, respectively 2013 for electric vehicles. The probabilistic density intervals show the probability that a quantity will be reached or exceeded if the diffusion follows current trends. The color gradient describes the quantiles of the projected capacities: the darker the color, the closer the capacity is to the median. The black dots show historical capacities and the black dotted lines, if visible, the maximum potential capacity that a country can install (see STAR methods in the main article). The red squares

and diamonds show required quantities for the energy transition, estimated in scenarios of European Commission[1,2] and Ten Year Network Development Plan[3] that are consistent with the European Green Deal policy package "Fit for 55", and national energy and climate policies (see STAR methods). Required quantities may be invisible if they are larger than the upper limit of the vertical axes of the projections. The Greek letters indicate a qualitative assessment on the suitability of the historical time series data for projecting probabilistic growth (see STAR methods), where a short historical diffusion (Θ) results in less reliable projections as empirical testing is comparatively short, decreasing historical diffusion rates (Δ) may result in early saturation, and a multi-S-curve pattern (Ψ) may lead to an underestimation of future growth. Figures S15-S24 provide projections for the remaining countries. Related to Figure 1.

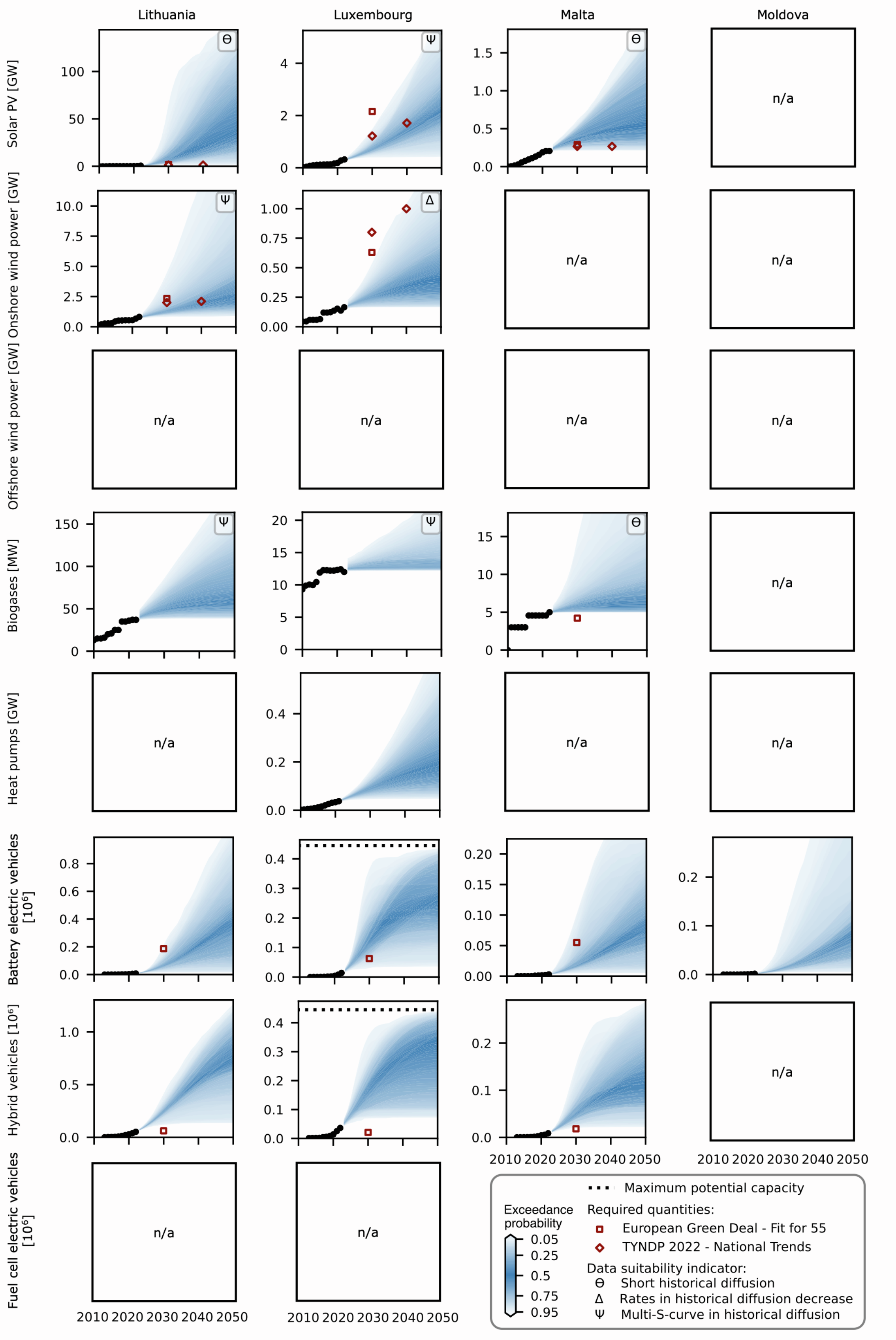

Figure S20. Probabilistic projections for all eight investigated technologies in Lithuania, Luxembourg, Malta, and Moldova, including training data until 2022, respectively 2021 for heat pumps. Training data starts in 1990, respectively 2013 for electric vehicles. The probabilistic density intervals show the probability that a quantity will be reached or exceeded if the diffusion follows current trends. The color gradient describes the quantiles of the projected capacities: the darker the color, the closer the capacity is to the median. The black dots show historical capacities and the black dotted lines, if visible, the maximum potential capacity that a country can install (see STAR methods in the main article). The red

squares and diamonds show required quantities for the energy transition, estimated in scenarios of European Commission[1,2] and Ten Year Network Development Plan[3] that are consistent with the European Green Deal policy package “Fit for 55”, and national energy and climate policies (see STAR methods). Required quantities may be invisible if they are larger than the upper limit of the vertical axes of the projections. The Greek letters indicate a qualitative assessment on the suitability of the historical time series data for projecting probabilistic growth (see STAR methods), where a short historical diffusion (Θ) results in less reliable projections as empirical testing is comparatively short, decreasing historical diffusion rates (Δ) may result in early saturation, and a multi-S-curve pattern (Ψ) may lead to an underestimation of future growth. Figures S15-S24 provide projections for the remaining countries. Related to Figure 1.

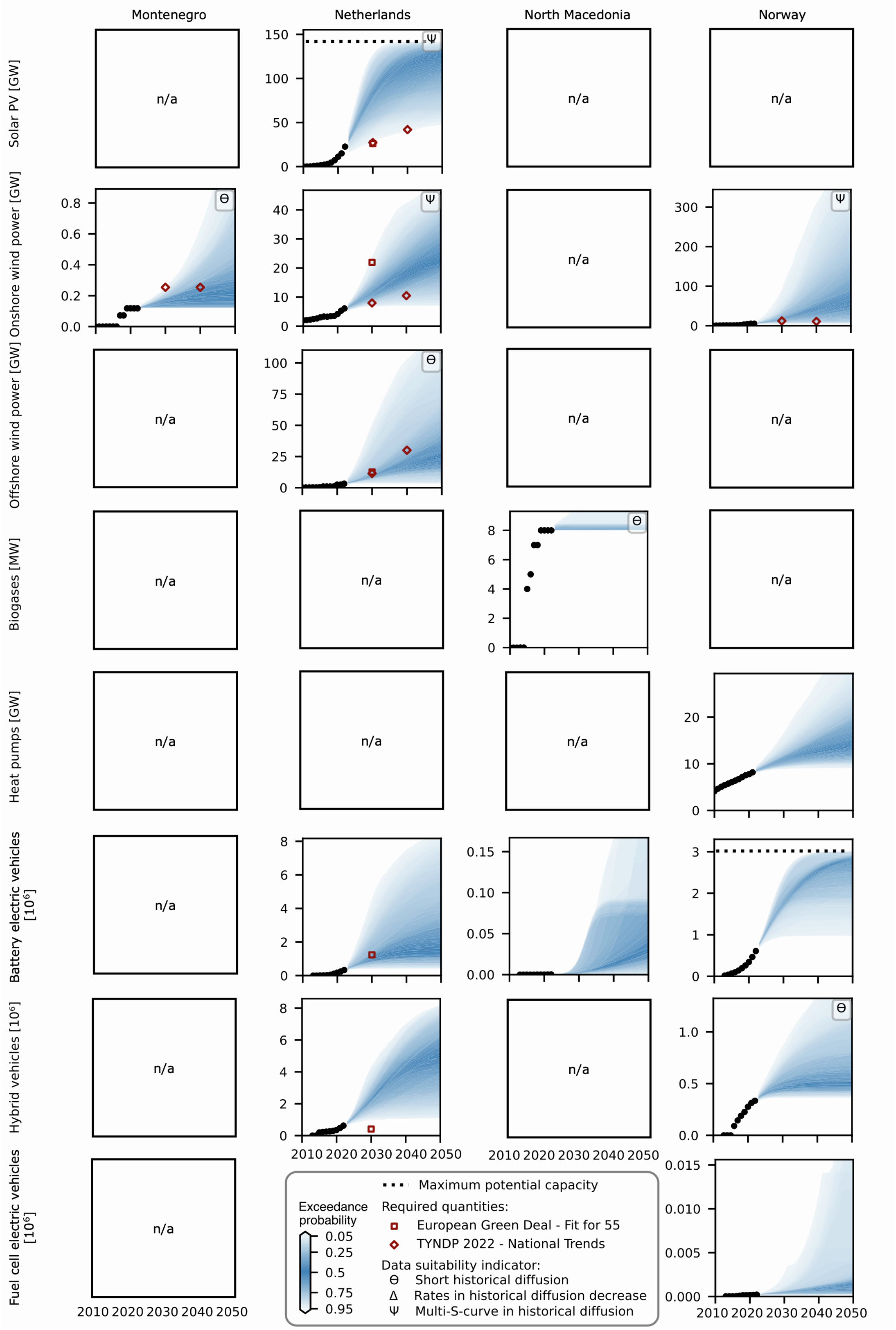

Figure S21. Probabilistic projections for all eight investigated technologies in Montenegro, Netherlands, North Macedonia, and Norway, including training data until 2022, respectively 2021 for heat pumps. Training data starts in 1990, respectively 2013 for electric vehicles. The probabilistic density intervals show the probability that a quantity will be reached or exceeded if the diffusion follows current trends. The color gradient describes the quantiles of the projected capacities: the darker the color, the closer the capacity is to the median. The black dots show historical capacities and the black dotted lines, if visible, the maximum potential capacity that a country can install (see STAR methods in the main article).

The red squares and diamonds show required quantities for the energy transition, estimated in scenarios of European Commission[1,2] and Ten Year Network Development Plan[3] that are consistent with the European Green Deal policy package “Fit for 55”, and national energy and climate policies (see STAR methods). Required quantities may be invisible if they are larger than the upper limit of the vertical axes of the projections. The Greek letters indicate a qualitative assessment on the suitability of the historical time series data for projecting probabilistic growth (see STAR methods), where a short historical diffusion (Θ) results in less reliable projections as empirical testing is comparatively short, decreasing historical diffusion rates (Δ) may result in early saturation, and a multi-S-curve pattern (Ψ) may lead to an underestimation of future growth. Figures S15-S24 provide projections for the remaining countries. Related to Figure 1.

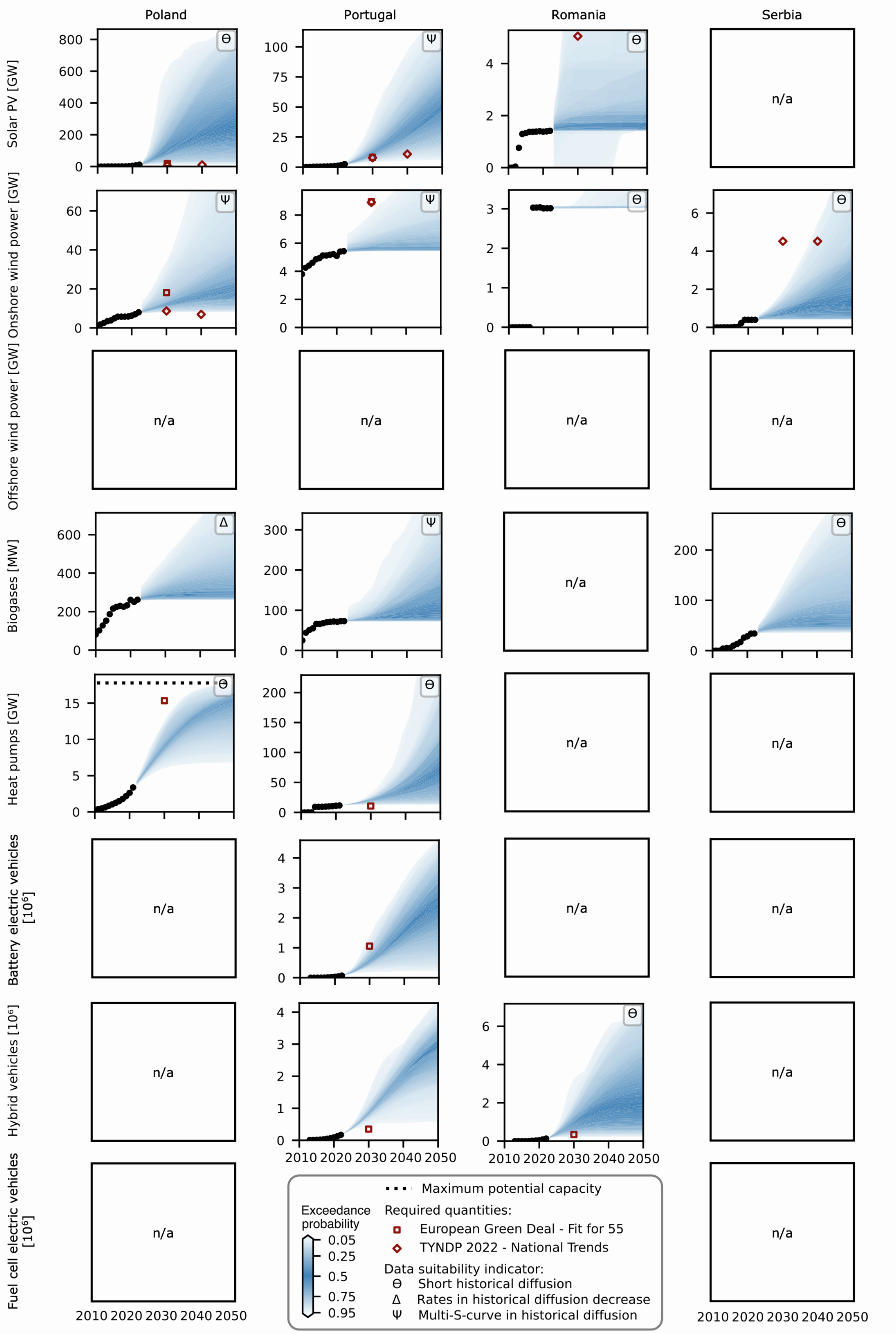

Figure S22. Probabilistic projections for all eight investigated technologies in Poland, Portugal, Romania, and Serbia, including training data until 2022, respectively 2021 for heat pumps. Training data starts in 1990, respectively 2013 for electric vehicles. The probabilistic density intervals show the probability that a quantity will be reached or exceeded if the diffusion follows current trends. The color gradient describes the quantiles of the projected capacities: the darker the color, the closer the capacity is to the median. The black dots show historical capacities and the black dotted lines, if visible, the maximum potential capacity that a country can install (see STAR methods in the main article). The red

squares and diamonds show required quantities for the energy transition, estimated in scenarios of European Commission[1,2] and Ten Year Network Development Plan[3] that are consistent with the European Green Deal policy package "Fit for 55", and national energy and climate policies (see STAR methods). Required quantities may be invisible if they are larger than the upper limit of the vertical axes of the projections. The Greek letters indicate a qualitative assessment on the suitability of the historical time series data for projecting probabilistic growth (see STAR methods), where a short historical diffusion (Θ) results in less reliable projections as empirical testing is comparatively short, decreasing historical diffusion rates (Δ) may result in early saturation, and a multi-S-curve pattern (Ψ) may lead to an underestimation of future growth. Figures S15-S24 provide projections for the remaining countries. Related to Figure 1.

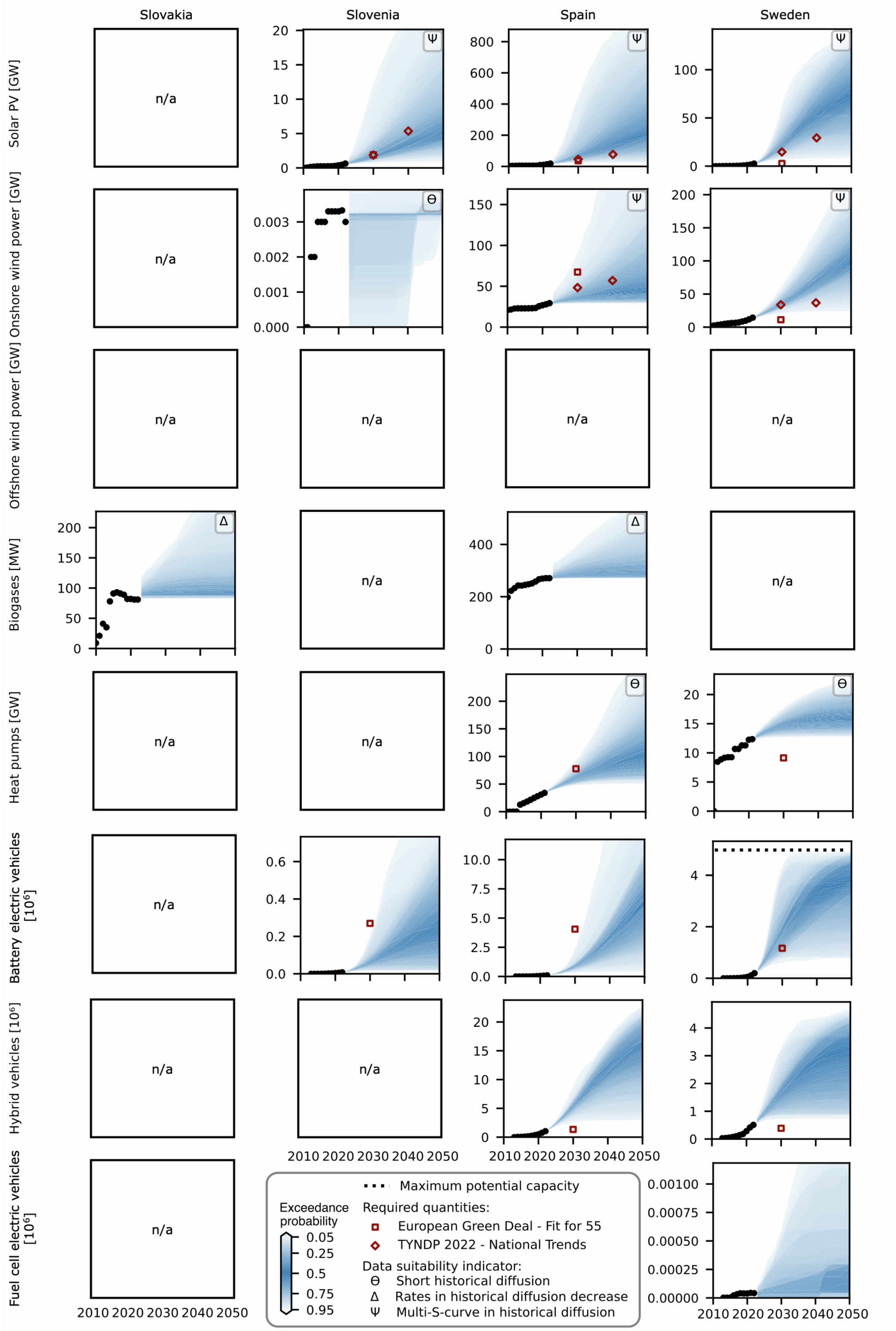

Figure S23. Probabilistic projections for all eight investigated technologies in Slovakia, Slovenia, Spain, and Sweden, including training data until 2022, respectively 2021 for heat pumps. Training data starts in 1990, respectively 2013 for electric vehicles. The probabilistic density intervals show the probability that a quantity will be reached or exceeded if the diffusion follows current trends. The color gradient describes the quantiles of the projected capacities: the darker the color, the closer the capacity is to the median. The black dots show historical capacities and the black dotted lines, if visible, the maximum potential capacity that a country can install (see STAR methods in the main article). The red squares

and diamonds show required quantities for the energy transition, estimated in scenarios of European Commission[1,2] and Ten Year Network Development Plan[3] that are consistent with the European Green Deal policy package “Fit for 55”, and national energy and climate policies (see STAR methods). Required quantities may be invisible if they are larger than the upper limit of the vertical axes of the projections. The Greek letters indicate a qualitative assessment on the suitability of the historical time series data for projecting probabilistic growth (see STAR methods), where a short historical diffusion (Θ) results in less reliable projections as empirical testing is comparatively short, decreasing historical diffusion rates (Δ) may result in early saturation, and a multi-S-curve pattern (Ψ) may lead to an underestimation of future growth. Figures S15-S24 provide projections for the remaining countries. Related to Figure 1.

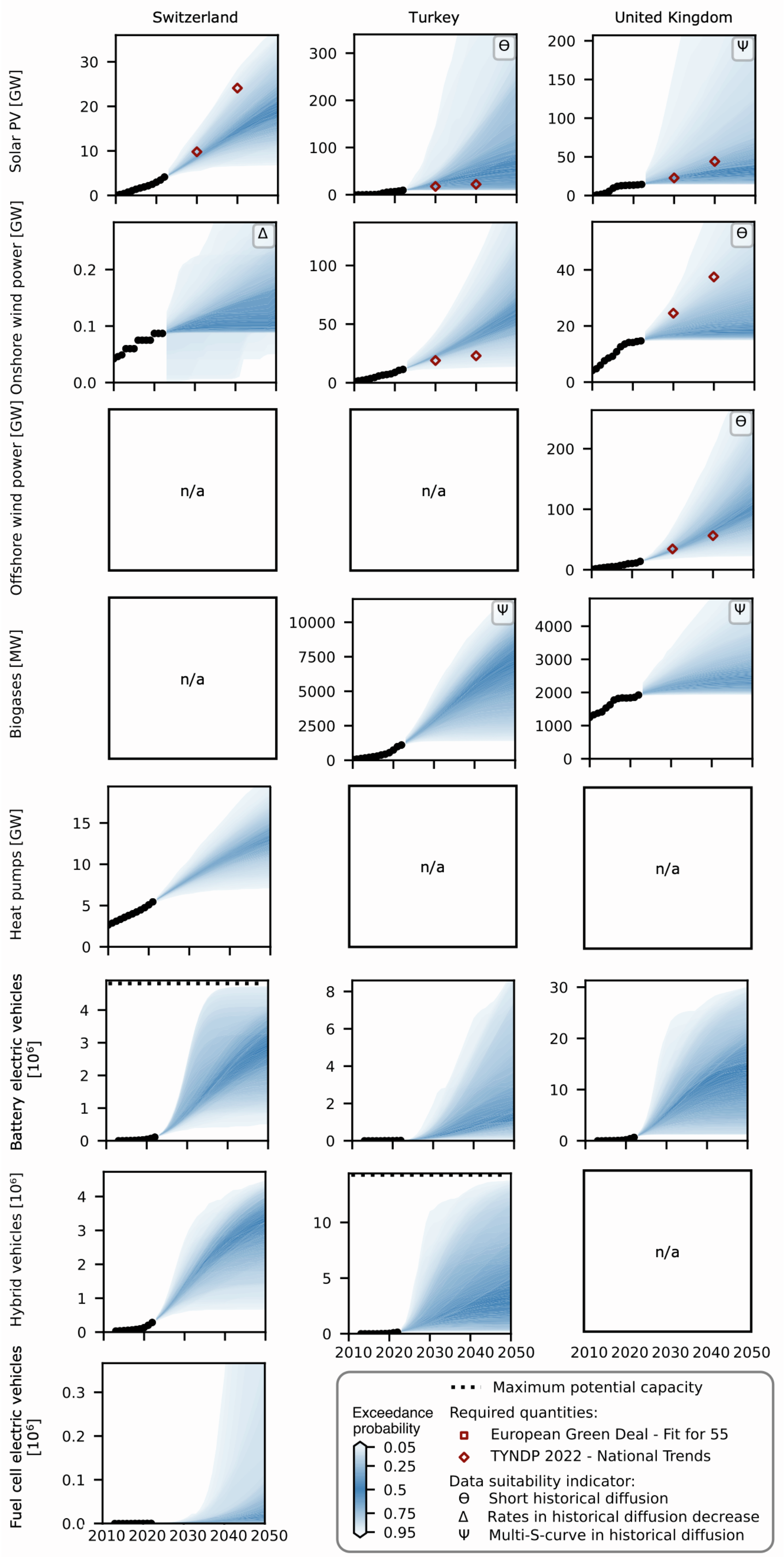

Figure S24. Probabilistic projections for all eight investigated technologies in Switzerland, Turkey, and the United Kingdom, including training data until 2022, respectively 2021 for heat pumps. Training data starts in 1990, respectively 2013 for electric vehicles. The probabilistic density intervals show the probability that a quantity will be reached or exceeded if the diffusion follows current trends. The color gradient describes the quantiles of the projected capacities: the darker the color, the closer the capacity is to the median. The black dots show historical capacities and the black dotted lines, if visible, the maximum potential capacity that a country can install (see STAR methods in the main article). The red

squares and diamonds show required quantities for the energy transition, estimated in scenarios of European Commission[1,2] and Ten Year Network Development Plan[3] that are consistent with the European Green Deal policy package “Fit for 55”, and national energy and climate policies (see STAR methods). Required quantities may be invisible if they are larger than the upper limit of the vertical axes of the projections. The Greek letters indicate a qualitative assessment on the suitability of the historical time series data for projecting probabilistic growth (see STAR methods), where a short historical diffusion (Θ) results in less reliable projections as empirical testing is comparatively short, decreasing historical diffusion rates (Δ) may result in early saturation, and a multi-S-curve pattern (Ψ) may lead to an underestimation of future growth. Figures S15-S23 provide projections for the remaining countries. Related to Figure 1.

Maps – Distribution of capacities across Europe

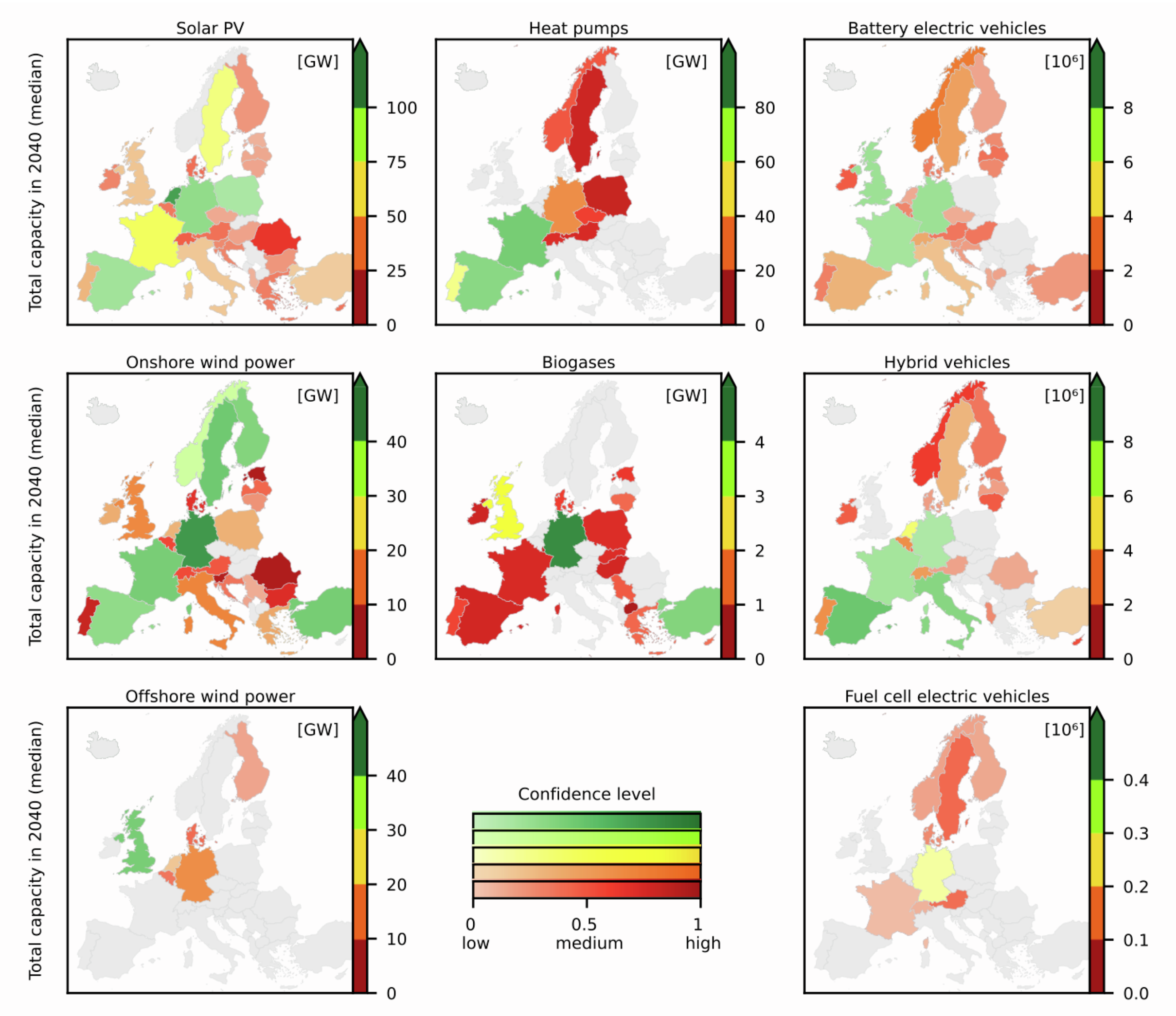

Figure S25. Distribution of median capacities of all eight investigated technologies across Europe in 2040. The colors group countries according to the total capacity estimated by the median in the probabilistic projections. The color gradient indicates the confidence level: the darker the color, the higher the confidence. The confidence level describes the width of a probabilistic projection and is defined as the share of quantiles that covers the range of ±25% from the projected median quantity (see STAR methods in the main article). The lower the share, the broader is the probabilistic projection. Countries in grey have no projection. See Figure 2 for the year 2030 and Figure S26 for 2050. See Figures S27-S29 for capacities per capita. Related to Figure 2.

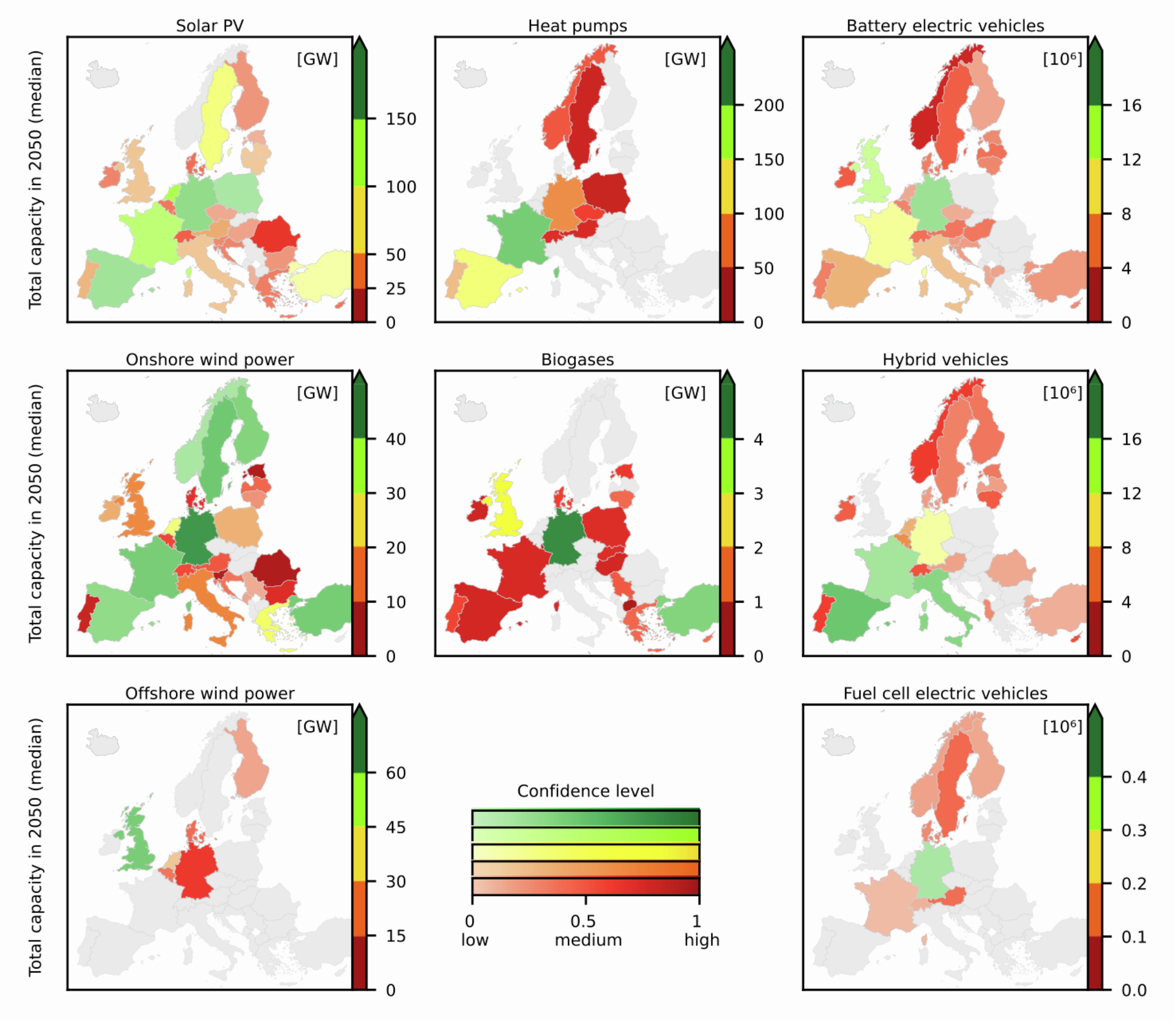

Figure S26. Distribution of median capacities of all eight investigated technologies across Europe in 2050. The colors group countries according to the total capacity estimated by the median in the probabilistic projections. The color gradient indicates the confidence level: the darker the color, the higher the confidence. The confidence level describes the width of a probabilistic projection and is defined as the share of quantiles that covers the range of ±25% from the projected median quantity (see STAR methods in the main article). The lower the share, the broader is the probabilistic projection. Countries in grey have no projection. See Figure 2 for the year 2030 and Figure S25 for 2040. See Figures S27-S29 for capacities per capita. Related to Figure 2.

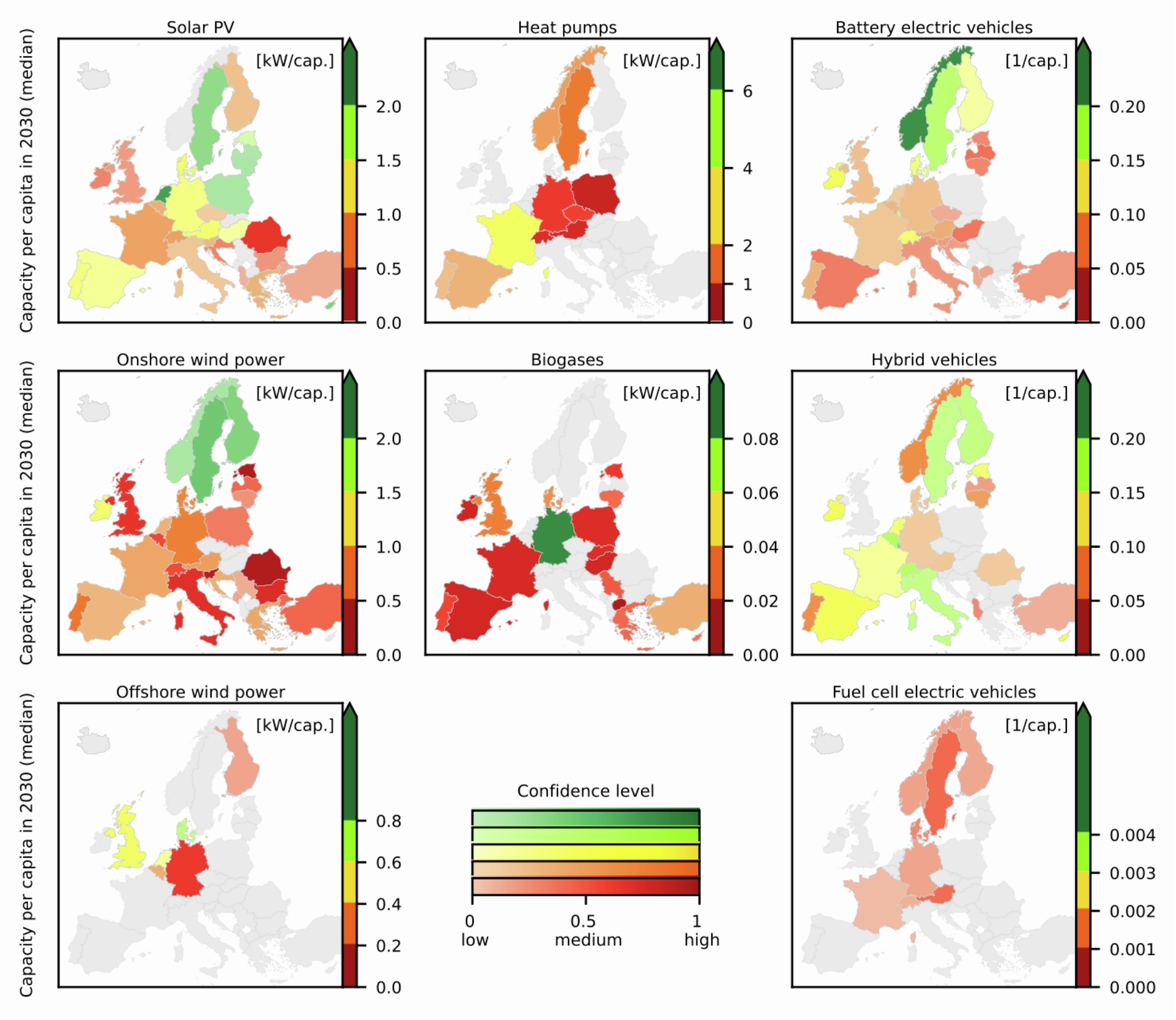

Figure S27. Distribution of median capacities per capita of all eight investigated technologies across Europe in 2030. The colors group countries according to the total capacity estimated by the median in the probabilistic projections. The color gradient indicates the confidence level: the darker the color, the higher the confidence. The confidence level describes the width of a probabilistic projection and is defined as the share of quantiles that covers the range of ±25% from the projected median quantity (see STAR methods in the main article). The lower the share, the broader is the probabilistic projection. Countries in grey have no projection. Capita numbers are taken from Eurostat[4] for the latest available year. See Figure S28 and Figure S29 for the years 2040 and 2050. Related to Figure 2.

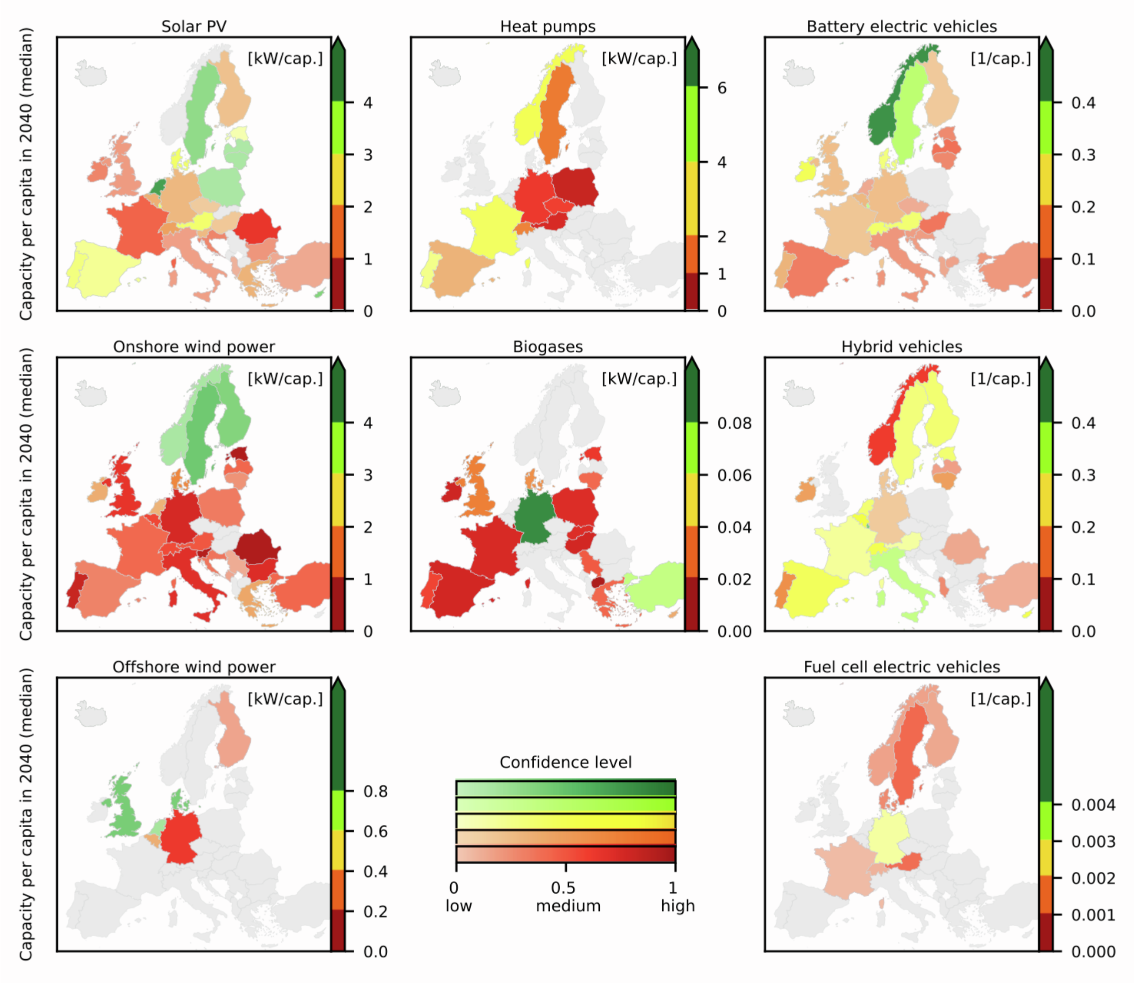

Figure S28. Distribution of median capacities per capita of all eight investigated technologies across Europe in 2040. The colors group countries according to the total capacity estimated by the median in the probabilistic projections. The color gradient indicates the confidence level: the darker the color, the higher the confidence. The confidence level describes the width of a probabilistic projection and is defined as the share of quantiles that covers the range of ±25% from the projected median quantity (see STAR methods in the main article). The lower the share, the broader is the probabilistic projection. Countries in grey have no projection. Capita numbers are taken from Eurostat[4] for the latest available year. See Figure S27 and Figure S29 for the years 2030 and 2050. Related to Figure 2.

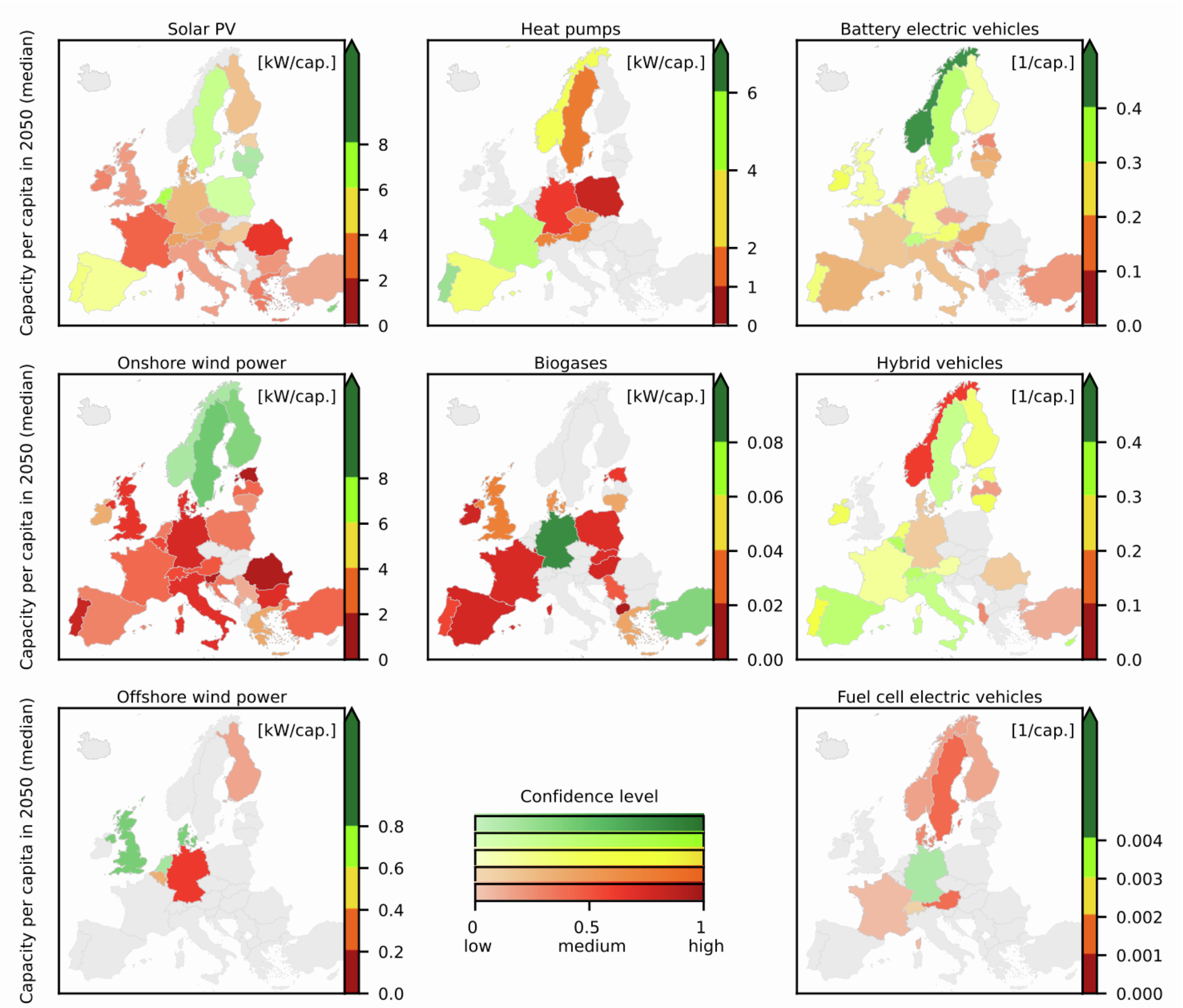

Figure S29. Distribution of median capacities per capita of all eight investigated technologies across Europe in 2050. The colors group countries according to the total capacity estimated by the median in the probabilistic projections. The color gradient indicates the confidence level: the darker the color, the higher the confidence. The confidence level describes the width of a probabilistic projection and is defined as the share of quantiles that covers the range of ±25% from the projected median quantity (see STAR methods in main article). The lower the share, the broader is the probabilistic projection. Countries in grey have no projection. Capita numbers are taken from Eurostat[4] for the latest available year. See Figure S27 and Figure S28 for the years 2030 and 2040. Related to Figure 2.

Maps – Probability of countries to reach required quantities

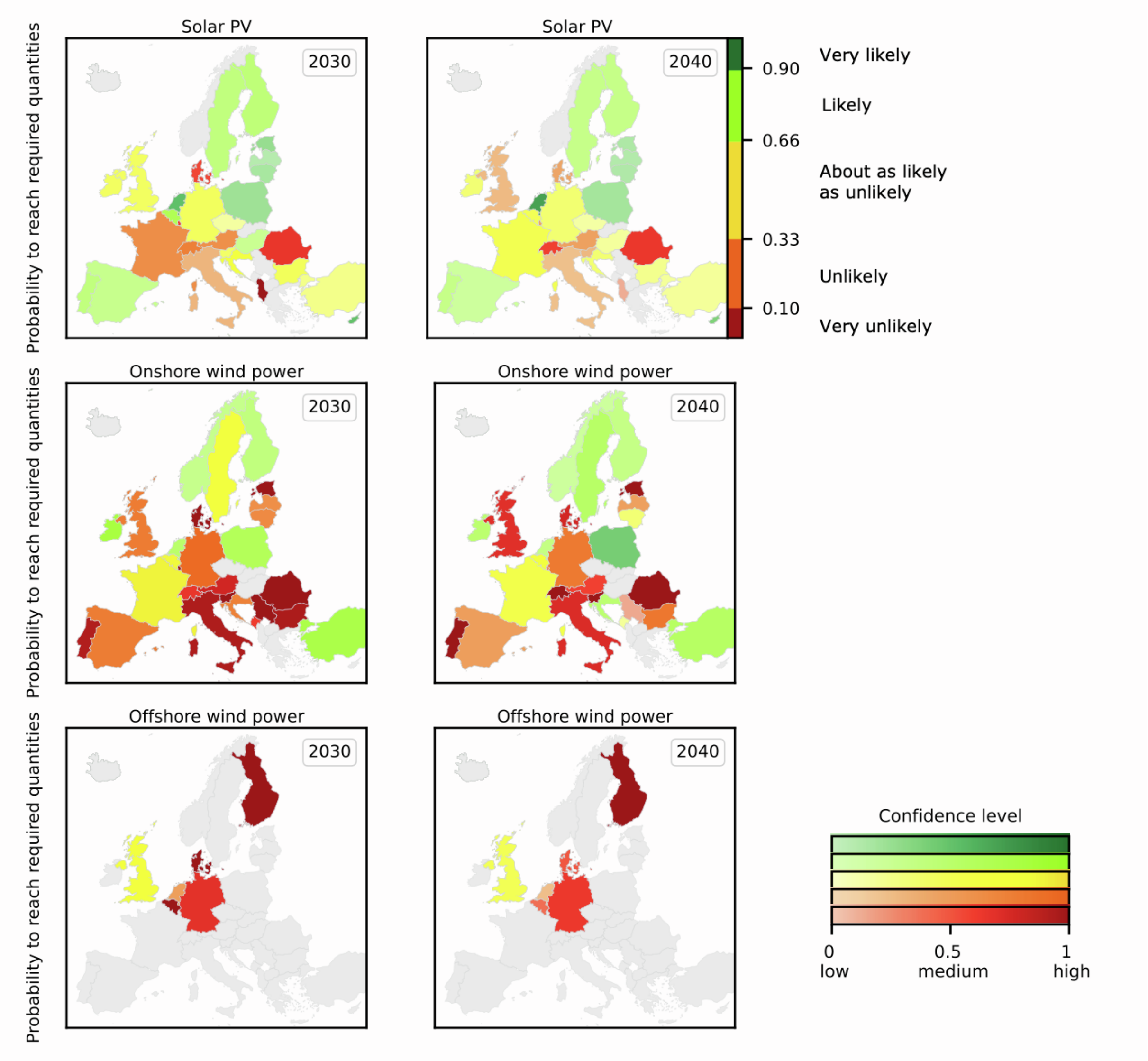

Figure S30. Map showing the exceedance probability of countries to reach required quantities in 2030 and 2040. The required quantities are estimated by the Ten Year Network Development Plan scenario "National Trends"[3] that is consistent with national energy and climate policies, and long-term strategies in line with European targets. The colors group all countries according to the level of probability and translate it into qualitative statements on likelihoods in line with earlier IPCC guidelines[5]. The likelihoods are conditional to the current dynamics and contextual conditions until the last training year. The color gradient indicates the confidence level: the darker the color, the higher the confidence. The confidence level describes the width of a probabilistic projection and is defined as the share of quantiles that covers the range of ±25% from the projected median quantity (see STAR methods in the main article). The lower the share, the broader is the probabilistic projection. Countries in grey have either no projection or no values of required quantities. Related to Figure 3.

Maps – Probability of countries to reach targets of national implementation reports

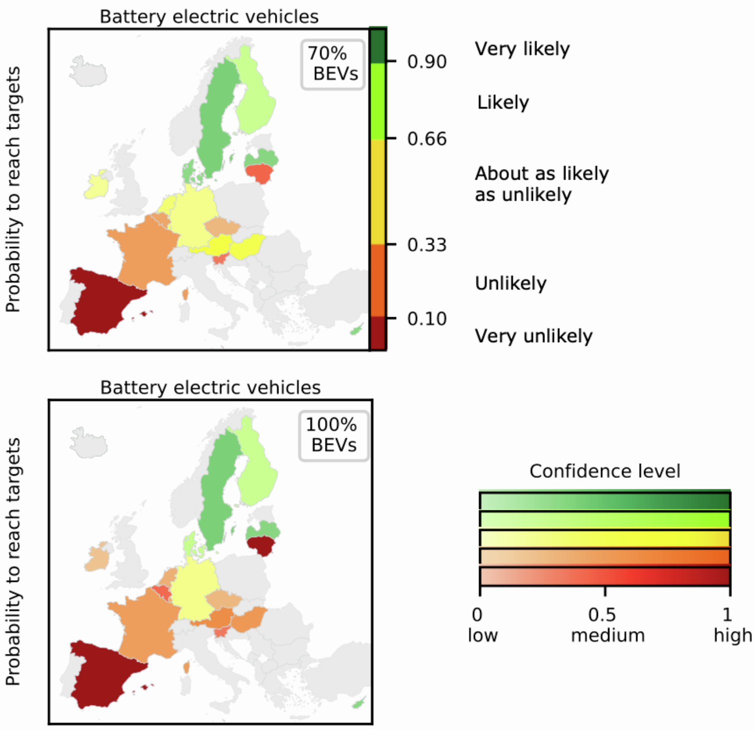

Figure S31. Map showing the exceedance probability of countries to reach targets of national implementation reports[6] corresponding to the Alternative Fuels Infrastructure Regulation[7] in 2030, based on the assumptions that the target values include a share of 70% of battery electric vehicles as estimated for European Commission[8] or a share of 100%. The colors group all countries according to the level of probability and translate it into qualitative statements on likelihoods in line with earlier IPCC guidelines[5]. The likelihoods are conditional to the current dynamics and contextual conditions until the last training year. The color gradient indicates the confidence level: the darker the color, the higher the confidence. The confidence level describes the width of a probabilistic projection and is defined as the share of quantiles that covers the range of ±25% from the projected median quantity (see STAR methods in the main article). The lower the share, the broader is the probabilistic projection. Countries in grey have either no projection or no values of required quantities. Related to Figure 3.

Supplemental tables

Table S1. Availability of historical time series data of technology capacities and required quantities for each country and if probabilistic projections are created until 2050 ("x" means yes).

Country	Solar PV			Wind onshore			Wind offshore			Biogases			Heat pumps			BEVs			Hybrid vehicles			FCEVs		
	Capacity	Projections	Required quantity	Capacity	Projections	Required quantity	Capacity	Projections	Required quantity	Capacity	Projections	Required quantity	Capacity	Projections	Required quantity	Capacity	Projections	Required quantity	Capacity	Projections	Required quantity	Capacity	Projections	Required quantity
Albania	x	x	x			x										x	x		x	x				
Austria	x	x	x	x	x	x						x	x	x	x	x	x	x	x	x	x	x	x	
Belgium	x	x	x	x	x	x	x	x	x	x		x			x	x	x	x	x	x	x			
Bosnia and Herzegovina	x	x	x	x		x				x	x					x			x					
Bulgaria	x	x	x	x	x	x				x		x			x	x		x	x		x			
Croatia	x	x	x	x	x	x				x		x			x	x	x	x			x	x		
Cyprus	x	x	x			x				x	x	x			x	x	x	x	x	x	x			
Czech Republic	x	x	x	x		x						x	x	x	x	x	x	x	x		x	x		
Denmark	x	x	x	x	x	x	x	x	x	x	x	x			x	x	x	x	x	x	x	x	x	
Estonia	x	x	x	x	x	x			x	x	x	x			x	x	x	x	x	x	x			
Finland	x	x	x	x	x	x	x	x	x			x			x	x	x	x	x	x	x	x	x	
France	x	x	x	x	x	x			x	x	x	x	x	x	x	x	x	x	x	x	x	x	x	
Georgia				x	x											x	x		x	x				
Germany	x	x	x	x	x	x	x	x	x	x	x	x	x	x	x	x	x	x	x	x	x	x	x	
Greece	x	x	x	x	x	x			x	x	x	x	x		x			x			x			
Hungary	x	x	x			x				x	x	x	x		x	x	x	x			x	x		
Iceland				x																				
Ireland	x	x	x	x	x	x			x	x	x	x			x	x	x	x	x	x	x			
Italy	x	x	x	x	x	x			x			x	x		x	x	x	x	x	x	x			
Latvia	x	x	x	x	x	x			x			x	x		x	x	x	x	x	x	x			
Liechtenstein																x	x		x	x				
Lithuania	x	x	x	x	x	x			x	x	x	x	x		x	x	x	x	x	x	x			
Luxembourg	x	x	x	x	x	x				x	x	x	x	x	x	x	x	x	x	x	x			
Malta	x	x	x			x				x	x	x			x	x	x	x	x	x	x			
Moldova																x	x							
Montenegro			x	x	x	x																		
Netherlands	x	x	x	x	x	x	x	x	x			x			x	x	x	x	x	x	x	x		
North Macedonia			x	x		x				x	x					x	x							
Norway	x		x	x	x	x			x				x	x		x	x		x	x		x	x	
Poland	x	x	x	x	x	x			x	x	x	x	x	x	x	x		x	x		x	x		
Portugal	x	x	x	x	x	x	x		x	x	x	x	x	x	x	x	x	x	x	x	x			
Romania	x	x	x	x	x	x				x		x				x		x	x	x	x			
Serbia			x	x	x	x				x														
Slovakia			x			x				x	x	x	x		x			x			x			
Slovenia	x	x	x	x	x	x						x			x	x	x	x			x			
Spain	x	x	x	x	x	x			x	x	x	x	x	x	x	x	x	x	x	x	x	x		
Sweden	x	x	x	x	x	x			x	x		x	x	x	x	x	x	x	x	x	x	x	x	
Switzerland	x	x	x	x	x	x							x	x		x	x		x	x		x	x	
Turkey	x	x	x	x	x	x				x	x					x	x		x	x				
United Kingdom	x	x	x	x	x	x	x	x	x	x	x					x	x							

Table S2. Probability that the final probabilistic projections of the weighted multi-model cover future observations with statistical significance (***p ≤ 0.001, **p ≤ 0.01, *p ≤ 0.05), calculated in binomial tests. Statistical significance is presented at probabilities of ≥ 0.95, otherwise for probabilities at p ≤ 0.05. Steps 3-4 in the Method details (STAR methods) in the main article describe the hindcasting procedure for calculating the out-of-interval counts. See Figures S7-S12 for the distribution of out-of-interval counts for all observations.

**Technology**	**Out-of-interval count** (number of tests, out-of-sample years used in testing)	**Number of observations falling within the density intervals of probabilistic projections** (=number of tests – out-of-interval count)	**Probability that probabilistic projections cover future observations with statistical significance**
Biogases	6 (n=262, 5 years)	256	0.95 **
Solar PV	0 (n=398, 5 years)	398	1 ***
Offshore wind power	1 (n=90, 5 years)	89	0.95 **
Onshore wind power	1 (n=362, 5 years)	361	0.95 ***
Heat pumps	0 (n=141, 5 years)	141	1 ***
Battery electric vehicles	7 (n=183, 3 years)	176	0.93 *
Hybrid cars	25 (n=136, 3 years)	111	0.76 *
Fuel cell electric vehicles	0 (n=39, 3 years)	39	1 ***

*Binomial test: ***p ≤ 0.001, **p ≤ 0.01, *p ≤ 0.05*

Supplemental methods

S1 Model variants to create probabilistic projections

Using the PROWIDE approach adapted from Zielonka et al.[9] and for each technology and country, we create probabilistic projections using 72 model variants that are a combination of one S-curve model (see S2), one curve fitting variant that uses Near-Optimal Differential Evolution (NODE) to create projections with and without the consideration of historical diffusion rates (NODE, NODE-RR, NODE-DR), and a historical interval length (5 years, 10 years, 15 years, and full history).

For each of the 72 model variants, we fit one S-curve model to the historical time series data of a technology in a country using differential evolution,[10,11] which randomly explores different parametrizations of the S-curve. S-curves describe the historically observed[12–14] technology diffusion of three phases (formation, exponential and stable growth, and saturation) using different parameters specific to each S-curve and including various contextual factors[15,16]. We repeat the curve fitting 225 or more times (depending on the variant) and, in each repetition, randomly select two parametrizations that the differential evolution explores until it finds an optimum parametrization for the S-curve that fits the data best. As doing so generates a collection of 450 or more deterministic projections around the optimum, we call this curve fitting method Near-Optimal Differential Evolution (NODE).

As curve fitting is prone to overfitting,[9,15] we use two additional variants of NODE that increase the width of the probabilistic projections in years of the near future. We do so by adding a data point to the end of the historical time series which is assumed to be the capacity of that year before we fit S-curves again. In the variant with Recent Rates (NODE-RR), we multiply each annual diffusion rate of the five most recent years of the historical time series with the capacity of the latest year to create five assumed capacities and eventually five different time series. In the variant with historical Diffusion of Rates (NODE-DR), we create capacities based on the last rate, and ±1 and ±2 standard deviations of all historical rates, centered around the latest rate.

In addition to fitting S-curves to the full historical time series, we limit the use of historical capacities to ranges of the most recent 5, 10, and 15 years. We do so since historical diffusion can show patterns of multiple S-curves that uniform S-curves may not represent properly and bi-S-curves are computationally expensive to fit and have a high risk to overfit or replicate the uniform curves.[9] As the historical diffusion of electric vehicles is comparatively short, we do not limit the historical interval length for such technologies.

S2 S-curves

We parametrize the six S-curves models of our study (Bass, Bertalanffy, Gompertz, logistic, Richards-4p, Richards-5p) using the same mathematical formulations as Zielonka et al.[9] (Equations (1)-(6)). Each S-curve model determines the installed capacity *f(t)* of a technology in year *t* with unitless model-specific parameters *p, q, k, d* and *b*, a time shift t_0 in years, and a level of saturation *(C-z)* of the same unit as *f(t)*. The vertical shift *z* allows a curve to start with capacities greater than zero.

Bass:

$$f(t) = (C - z) \cdot \frac{1 - \exp\big(-(p+q)(t-t_0)\big)}{1 + \frac{q}{p} \cdot \exp\big(-(p+q)(t-t_0)\big)} + z \quad (1)$$

Bertalanffy:

$$f(t) = (C - z) \cdot \big(1 - b \cdot \exp\big(-\mathrm{k} \cdot (\mathrm{t} - t_0)\big)\big)^3 + z \quad (2)$$

Gompertz:

$$f(t) = (C - z) \cdot \exp\big(-\exp\big(-k \cdot (t - t_0)\big)\big) + z \quad (3)$$

Logistic:

$$f(t) = \frac{C - z}{1 + \exp\left(-k \cdot (t - t_0)\right)} + z \quad (4)$$

Richards-4p:

$$f(t) = (C - z) \cdot \left(1 - \frac{1}{d} \cdot \exp\big(-\mathrm{k} \cdot (\mathrm{t} - t_0)\big)\right)^d + z \quad (5)$$

Richards-5p:

$$f(t) = (C - z) \cdot \left(1 - b \cdot \exp\left(-\mathrm{k} \cdot (\mathrm{t} - t_0)\right)\right)^d + z \tag{6}$$

To reduce computational complexity, we limit the parameters using the same bounds as Zielonka et al.[9] :

Table S3. Parameter bounds of S-curves.

Parameter(s)	Lower bound	Upper bound
C	Last value in time series used for curve fitting	Technical potential of a technology in a country
t_0	Bass, Gompertz, logistic: 2000<br>Bertalanffy, Richards: 1900	Bass, Gompertz, logistic: 2100<br>Bertalanffy, Richards: 2100
d	0	10
z	0	First value in time series used for curve fitting
b, k, p, q	0	1

S3 Data on maximum potential capacities

To limit the potential of maximum installable capacities we use different statistical data from literature and Eurostat. To limit the influence of our choice on the projections, we select relatively high values. For solar PV and wind, we use the upper quartile of potentials provided across literature[17]. For biogases, we assume that all national capacities can increase by a factor of 15 to give enough space to grow as the European Biogas Association[18] estimates an increase in production of at least fivefold by 2050. For other power generation technologies that have a historical time series but no potential in some countries (e.g., Albania, Malta, and Turkey for solar PV, and Montenegro, Serbia, and Turkey for onshore wind power), we add the average ratio between potential and currently installed capacity of all countries. For heat pumps, we assume that the share of heat pumps in space heating can increase to 100%. Accordingly, we approximate the resulting potentials by dividing the current capacities by the current share of heat in space heating given by Eurostat[19], IDEA[20], and SEAI[21] and assume that a future increase in the share is equivalent to an increase in heat pump capacity of the same magnitude. For Cyprus, Luxembourg, and Malta, we assume current shares of heat pumps at 1%. For electric vehicles, we set the limit to the current total number of passenger cars in each country.[22]

Supplemental references

1. European Commission (2021). Policy scenarios for delivering the European Green Deal. https://energy.ec.europa.eu/data-and-analysis/energy-modelling/policy-scenarios-delivering-european-green-deal_en.

2. European Commission (2024). Climate Target Plan 2040 Impact Assessment. https://climate.ec.europa.eu/eu-action/climate-strategies-targets/2040-climate-target_en#documents.

3. ENTSOG, and ENTSO-E (2022). TYNDP 2022 Scenario Report (Version April 2022). https://2022.entsos-tyndp-scenarios.eu/wp-content/uploads/2022/04/TYNDP2022_Joint_Scenario_Full-Report-April-2022.pdf.

4. Eurostat (2023). Population on 1 January (version 27-11-2023). EC data browser. https://ec.europa.eu/eurostat/databrowser/view/tps00001/default/table?lang=en.

5. Frame, D.J., Held, H., Kriegler, E., Mach, K.J., Matschoss, P.R., Plattner, G.-K., Zwiers, F.W., and Matschoss, P.R. (2010). Guidance Note for Lead Authors of the IPCC Fifth Assessment Report on Consistent Treatment of Uncertainties (Intergovernmental Panel on Climate Change (IPCC)).

6. European Commission (2024). European Alternative Fuels Observatory - Target tracker. https://alternative-fuels-observatory.ec.europa.eu/transport-mode/road/european-union-eu27/target-tracker.

7. European Parliament, and European Council (2023). Regulation (EU) 2023/1804 of the European Parliament and of the Council of 13 September 2023 on the deployment of alternative fuels infrastructure, and repealing Directive 2014/94/EU.

8. European Commission (2021). EU Reference Scenario 2020. https://energy.ec.europa.eu/data-and-analysis/energy-modelling/eu-reference-scenario-2020_en.

9. Zielonka, N., Wen, X., and Trutnevyte, E. (2023). Probabilistic projections of granular energy technology diffusion at subnational level. PNAS Nexus *2*, pgad321. https://doi.org/10.1093/pnasnexus/pgad321.

10. Storn, R., and Price, K. (1997). Differential Evolution – A Simple and Efficient Heuristic for Global Optimization over Continuous Spaces. Journal of Global Optimization, 341–359. https://doi.org/10.1023/A:1008202821328.

11. Phillips, J. Raman Spectroscopy Fit. Available at https://bitbucket.org/zunzuncode/ramanspectroscopyfit/src/master/. Deposited 28 July 2018. Bitbucket.

12. Odenweller, A., Ueckerdt, F., Nemet, G.F., Jensterle, M., and Luderer, G. (2022). Probabilistic feasibility space of scaling up green hydrogen supply. Nat Energy *7*, 854–865. https://doi.org/10.1038/s41560-022-01097-4.

13. Wilson, C., Grubler, A., Bauer, N., Krey, V., and Riahi, K. (2013). Future capacity growth of energy technologies: are scenarios consistent with historical evidence? Climatic Change *118*, 381–395. https://doi.org/10.1007/s10584-012-0618-y.

14. Geroski, P.A. (2000). Models of technology diffusion. Research Policy *29*, 603–625. https://doi.org/10.1016/S0048-7333(99)00092-X.

15. Höök, M., Li, J., Oba, N., and Snowden, S. (2011). Descriptive and Predictive Growth Curves in Energy System Analysis. Nat Resour Res *20*, 103–116. https://doi.org/10.1007/s11053-011-9139-z.

16. Lekvall, P., and Wahlbin, C. (1973). A Study of Some Assumptions Underlying Innovation Diffusion Functions. The Swedish Journal of Economics *75*, 362. https://doi.org/10.2307/3439146.

17. Dupré La Tour, M.-A. (2023). Photovoltaic and wind energy potential in Europe – A systematic review. Renewable and Sustainable Energy Reviews *179*, 113189. https://doi.org/10.1016/j.rser.2023.113189.

18. European Biogas Association (2023). Biogas & biomethane in a nutshell. https://www.europeanbiogas.eu/benefits/#growth-potential.

19. Eurostat (2023). Energy consumption in households (data 2021). Eurostat Statistics Explained. https://ec.europa.eu/eurostat/statistics-explained/index.php?title=Energy_consumption_in_households#Energy_products_used_in_the_residential_sector.

20. Instituto para la Diversificación y Ahorro de la Energia (IDEA) (2022). Consumos del Sector Residencial en España - Resumen de Información Básica. https://www.idae.es/uploads/documentos/documentos_Documentacion_Basica_Residencial_Unido_c93da537.pdf.

21. Sustainable Energy Authority of Ireland (SEAI) (2023). Residential - Final energy by end-use. https://www.seai.ie/data-and-insights/seai-statistics/key-statistics/residential/.

22. Eurostat (2023). Passenger cars, by type of motor energy (version 29-11-2023). EC data browser. https://ec.europa.eu/eurostat/databrowser/view/road_eqs_carpda/default/table?lang=en.
